# Graph augmented transformers improve chemotherapy toxicity symptom extraction from clinical notes

**DOI:** 10.1038/s41467-026-72347-2

**Published:** 2026-04-28

**Authors:** Elia Saquand, Behzad Naderalvojoud, Maximilian Schuessler, Malvika Pillai, Brian Travis Rice, Douglas W. Blayney, Tina Hernandez-Boussard

**Affiliations:** 1 Department of Medicine, Stanford University, Stanford, CA USA; 2 Department of Biomedical Data Science, Stanford University, Stanford, CA USA; 3 Department of Emergency Medicine, Stanford University, Stanford, CA USA; 4 Department of Surgery, Stanford University, Stanford, CA USA

**Keywords:** Translational research, Diagnosis, Outcomes research

## Abstract

Chemotherapy is essential for cancer treatment but may cause adverse events requiring emergency department visits and hospitalizations, placing substantial burdens on patients and healthcare systems. Existing approaches to detect these events often rely on structured electronic health records (EHR) data, which incompletely capture patients’ symptom trajectories. Clinical notes contain richer information yet remain challenging to synthesize. Here we show that integrating transformer-based language models with graph neural networks improves extraction of chemotherapy-related toxicity symptoms from clinical notes. We developed Graph-Augmented Transformer for Clinical Notes (GAT-CN), which embeds patient notes using Bio+ClinicalBERT and links them to symptom-related terms within a heterogeneous clinical graph learned using GraphSAGE. In a multi-symptom classification task, GAT-CN outperformed transformer-only models, achieving a weighted AUROC of 0.850 and AUPRC of 0.812. The model also identified additional diagnoses not captured in structured EHRs, confirmed through manual annotation. These results demonstrate that graph-augmented models improve symptom detection from clinical narratives and support earlier monitoring of chemotherapy-related adverse events.

## Abstract (web summary)

Adverse events from chemotherapy are common; however, identifying related symptoms following these events from clinical documentation can be challenging. Here, the authors develop a natural language processing model to extract symptoms from clinical notes.

## Introduction

Systemic antineoplastic therapy, such as chemotherapy, remains a cornerstone in the fight against cancer but often results in adverse events^1^. For example, patients can experience debilitating pain, vomiting, and potentially life-threatening infections with sepsis^2,3^. These adverse outcomes often lead to post-chemotherapy emergency department (ED) visits or inpatient hospital admissions, collectively described as acute care utilization (ACU) events^4,5^, which are both costly and detrimental to patients’ quality of life^6^. Reported ACU rates vary across cancers and settings: for example, 47% of breast cancer patients experience an ED visit or hospitalization within 180 days of adjuvant chemotherapy^7^, while another study found 11.9% hospitalized and 17.1% visiting the ED^8^. Moreover, more than one-third of primary care visits during chemotherapy are for treatment-related side effects, which nearly double the risk of subsequent ED visits or hospitalizations^9^. Together, these examples underscore the substantial burden of chemotherapy-related adverse events on patients and healthcare systems. Reflecting this burden, the Centers for Medicare & Medicaid Services (CMS) established the OP-35 quality measure^10^, which defines ACU within 30 days of chemotherapy as an indicator of poor quality of care and seeks to reduce preventable diagnoses^11^.

Artificial Intelligence (AI) is transforming healthcare at an unprecedented pace. From predictive analytics to diagnostic tools, AI is now deeply embedded across the continuum of care. Its applications span clinical decision support, patient monitoring, and operational efficiencies within healthcare systems. In oncology, AI tools are increasingly used to predict the risk of preventable ACU events following treatment initiation^12,13^. Many of these tools rely on structured electronic health record (EHR) data, such as diagnostic and procedure codes, which are often incomplete and noisy, limiting their accuracy ^14–16^. These concerns about data quality, combined with challenges related to model validation, clinical integration, and regulatory approval, affect the performance and widespread deployment of these tools in healthcare settings^17^. Without symptom monitoring, predicting ACU events from patient history is challenging. As a result, healthcare systems remain largely reactive, addressing ACU events only after they occur rather than proactively preventing them.

**Fig. 1:**
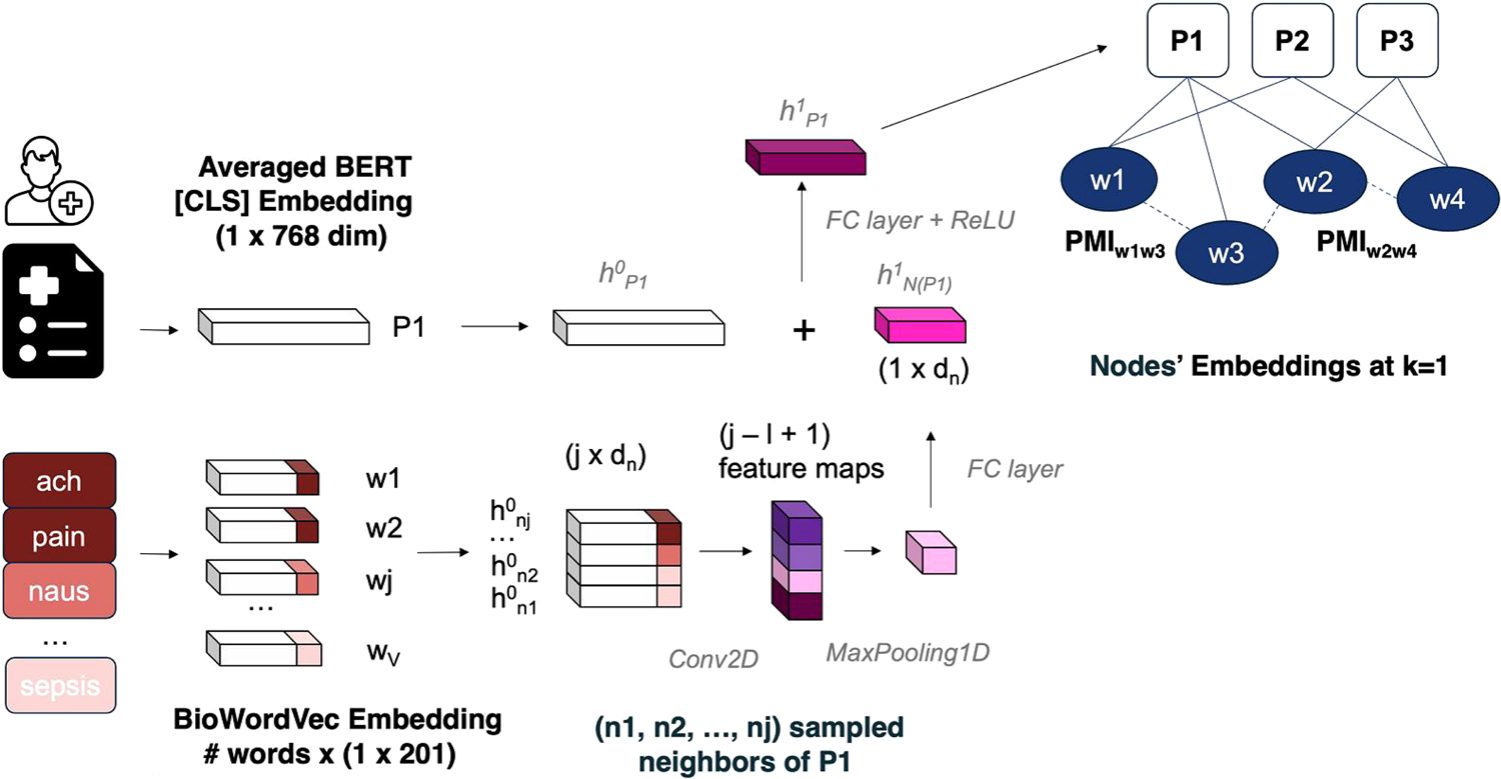
Overview of the GAT-CN architecture at depth *k* = 1. Each patient-note node (${P}_{1}$) is initialized with a 768-dimensional Bio+ClinicalBERT embedding (${h}_{{P}_{1}}^{0}$). During the first GraphSAGE message-passing layer ($k=1$), this embedding is updated to ${h}_{{P}_{1}}^{(1)}$ by integrating the aggregated representations of its immediate neighbor diagnosis-term nodes. Each diagnosis-term node combines a 200-dimensional BioWordVec embedding with a one-dimensional diagnosis-symptom class encoding, yielding a 201-dimensional representation. The 1-dimensional diagnosis-symptom class encoding serves as a lightweight indicator of curated symptom categories, while semantic information is primarily captured by the BioWordVec embeddings and graph-based aggregation. Neighbor embeddings are processed through a convolutional layer, followed by 1D max-pooling and a fully connected (FC) layer to obtain the aggregated neighbor representation ${h}_{{{{\mathcal{N}}}}({P}_{1})}^{(1)}$. The patient-note embedding is then refined through a ReLU-activated FC layer to yield the updated representation. For deeper layers ($k > 1$), the same aggregation and update procedure is recursively applied to propagate information across the heterogeneous graph.

To address these limitations and more accurately capture post-chemotherapy symptoms documented during routine care, we introduce Graph-Augmented Transformer for Clinical Notes (GAT-CN)—a framework that integrates ClinicalBERT^18^, a transformer encoder pretrained on clinical text, with graph neural networks (GNNs) (Fig. 1). The transformer encoder captures rich contextual representations of clinical narratives, enabling a nuanced understanding of symptom mentions within patient notes available prior to downstream acute care events. These representations are incorporated into a heterogeneous clinical graph, where nodes represent patient notes and symptom terms derived from an external symptom-class vocabulary, and edges encode contextual and symptom-specific relationships. Using the inductive GraphSAGE algorithm^19^, GAT-CN learns aggregation functions that generate node embeddings from neighboring nodes, allowing the model to generalize to new patient notes without reconstructing the entire graph. This integration of contextual language understanding with structured symptom knowledge enables GAT-CN to uncover implicitly documented or contextually linked toxicity symptoms, which may precede ACU, advancing early detection and supporting proactive monitoring and management of chemotherapy-related toxicity symptoms rather than direct prediction of ACU events.

In this work, we develop and evaluate GAT-CN for extracting chemotherapy-related toxicity symptoms from clinical notes and compare its performance with traditional and transformer-only models. We show that incorporating symptom relationships within a heterogeneous clinical graph improves multi-symptom detection compared with transformer-based models alone. The model also identifies clinically relevant diagnoses not captured in structured EHR data, confirmed through manual annotation. These findings demonstrate that graph-augmented transformer models enhance symptom detection from clinical narratives and may support earlier identification and management of chemotherapy-related adverse events in oncology care.

## Results

The target cohort comprised 1753 patients who received acute care within 30 days of initiation of chemotherapy due to a diagnosis listed under the OP-35 criterion and had at least one clinical note. Among these patients, 1140 (65%) were diagnosed with pain, 773 (44%) with diarrhea, vomiting, nausea, or dehydration (DVND), 574 (33%) with anemia, and 884 (50%) with neutropenia, pneumonia, fever, or sepsis (NPFS). As described in the Methods, these four groups correspond to the ten OP-35 diagnoses, which were aggregated into clinically actionable categories for analysis. Among the 1753 patients who experienced at least one ACU event within 30 days of chemotherapy initiation, 401 had an ED visit without subsequent admission, 1257 were admitted as inpatients, and 95 experienced both ED and inpatient during the observation period. On average, patients had two clinical notes and were assigned two OP-35 diagnoses.

### Cohort study

The cohort consisted of a diverse population with almost equal gender representation, with a mean age of 56.9 (SD = 15.8). Hematopoietic tumors (*n* = 377, 21.5%) were the most prevalent cancer type, followed by hepatobiliary pancreas (*n* = 203, 11.6%), gastrointestinal (*n* = 181, 10.3%), breast (*n* = 191, 10.9%), and lung (*n* = 184, 10.5%) cancers. These five types accounted for approximately two-thirds of the dataset. Lymphoma tumor patients were more likely to be diagnosed with anemia (*n* = 166, 28.9%). Lung cancer patients were more likely to be diagnosed with neutropenia, pneumonia, fever, or sepsis (*n* = 117, 13.2%) (Table 1). The classification of cancer types into the 10 cancer groups is available in Supplementary Table 1.

**Table 1:** Patient characteristics stratified by post-chemotherapy diagnosis categories

Patient characteristic	Total	Pain	Nausea, dehydration, diarrhea, vomiting	Anemia	Neutropenia, fever, pneumonia, sepsis
	(*n* = 1753)	(*n* = 1140, 65%)	(*n* = 773, 44%)	(*n* = 574, 33%)	(*n* = 884, 50%)
Age, mean ± SD	56.9 ± 15.8	56.4 ± 15.5	56.3 ± 15.8	56.0 ± 16.1	57.6 ± 15.7
Sex, *n* (%)					
Female	870 (49.6)	600 (52.6)	433 (56.0)	265 (46.2)	426 (48.2)
Male	883 (50.4)	540 (47.4)	340 (44.0)	309 (53.8)	458 (51.8)
Race, *n* (%)					
White	854 (48.7)	536 (47.1)	369 (47.8)	279 (48.7)	426 (48.2)
Asian	418 (23.9)	275 (24.1)	178 (23.1)	120 (20.9)	225 (25.5)
Black	67 (3.8)	46 (4.0)	30 (3.9)	23 (4.0)	35 (4.0)
Other	413 (23.6)	282 (24.8)	195 (25.3)	151 (26.4)	197 (22.3)
Ethnic group, *n* (%)					
Hispanic	283 (16.2)	201 (17.6)	130 (16.8)	107 (18.7)	126 (14.3)
Non-Hispanic	1458 (83.2)	932 (81.8)	637 (82.5)	459 (80.1)	752 (85.2)
Cancer group, *n* (%)					
Hematopoietic lymph	377 (21.5)	211 (18.5)	146 (18.9)	166 (28.9)	213 (24.1)
Pancreas	203 (11.6)	156 (13.7)	95 (12.3)	61 (10.6)	102 (11.5)
Gastrointestinal	181 (10.3)	122 (10.7)	103 (13.3)	48 (8.4)	82 (9.3)
Breast	191 (10.9)	127 (11.1)	89 (11.5)	38 (6.6)	100 (11.3)
Lung thoracic	184 (10.5)	119 (10.4)	59 (7.6)	57 (9.9)	117 (13.2)
Gynecologic	123 (7.0)	95 (8.3)	70 (9.1)	35 (6.1)	45 (5.1)
Head/Neck	136 (7.8)	80 (7.0)	56 (7.2)	40 (7.0)	68 (7.7)
Genitourinary	131 (7.5)	85 (7.5)	53 (6.9)	45 (7.8)	60 (6.8)
Sarcoma	117 (6.7)	77 (6.8)	56 (7.2)	53 (9.2)	50 (5.7)
Skin	46 (2.6)	33 (2.9)	21 (2.7)	13 (2.3)	17 (1.9)
Cancer stage, *n* (%)					
Stage 0–I	176 (18.5)	110 (18.8)	80 (19.9)	53 (20.0)	90 (18.0)
Stage II	168 (17.7)	106 (18.1)	81 (20.1)	42 (15.8)	82 (16.4)
Stage III	167 (17.6)	104 (17.7)	70 (17.4)	40 (15.1)	81 (16.2)
Stage IV	438 (46.2)	266 (45.4)	172 (42.7)	130 (49.1)	247 (49.4)
Insurance (%)					
Medicare	437 (57.1)	265 (54.9)	188 (57.1)	147 (57.0)	230 (59.0)
Private	257 (33.6)	167 (34.6)	115 (35.0)	87 (33.7)	128 (32.8)
Medicaid	71 (9.3)	51 (10.6)	26 (7.9)	24 (9.3)	32 (8.2)

Continuous variables are presented as means ± standard deviation (SD), and categorical variables as counts (*n*) and percentages (%). Column titles indicate the total number of patients in each category, along with their percentage relative to the total cohort.

### Diagnosis vocabulary and note annotation

The vocabulary creation process generated 207 diagnostic-related terms, categorized into four classes: 52 terms for Pain, 50 for DVND, 25 for Anemia, and 80 for NPFS. Examples of words included in each class can be found in Supplementary Table 2. Following the preprocessing steps, we obtained 1753 aggregated notes, each representing a patient in the cohort. The dataset was divided into three separate sets: training with 1317 patients, validation with 330 patients, and testing with 106 patients. While the training and validation sets were labeled using patient OP-35 diagnosis codes in the EHR data, the 106 patient notes used in the test set were manually annotated by four annotators. Although diagnosis code-based labels were also available for the test set, we intentionally relied on manual annotation by four independent annotators for the held-out test data to establish a higher-fidelity reference standard. This annotation also captures symptoms that may be absent or incompletely represented in the structured EHR data, enabling more robust evaluation. The overall inter-annotator agreement (IAA) across the test set varied by class, with perfect agreement for NPFS (IAA = 1.0), fair agreement for Pain (0.59) and DNVD (0.49), and lower agreement for Anemia (0.26). Detailed pairwise IAA scores for each diagnostic class are provided in Supplementary Table 3.

### Model performance

We evaluated model performance using two complementary approaches: (1) class-based metrics, which assess discrimination and predictive performance for each OP-35 symptom class individually, and (2) multi-label metrics, which account for the fact that a single patient note may contain multiple simultaneous symptom classes. Using class-based metrics, GAT-CN outperformed all other models in both weighted average AUROC and AUPRC, as shown in Table 2. Specifically, it achieved the highest AUROC (0.850, 95% CI: 0.833–0.867) and AUPRC (0.812, 95% CI: 0.791–0.831), exceeding state-of-the-art transformer models such as BioMed RoBERTa, Bio+ClinicalBERT, and Longformer, as well as the Random Forest baseline. To assess the model’s performance across individual diagnostic classes, Supplementary Fig. 1 displays the ROC and PR curves for GAT-CN. The weighted average precision, recall, and F1 scores are reported in Supplementary Table 4. The rule-based dictionary-matching baseline achieved a high recall of 0.99 but a low precision of 0.505. In contrast, GAT-CN achieved a significantly higher precision of 0.780 (*P* < 0.001).

**Table 2:** Model performance across evaluation metrics

Model	AUROC ↑ (95% CI)	AUPRC ↑ (95% CI)	HL ↓ (95% CI)	SA ↑ (95% CI)
Random Forest+TF-IDF	0.735 (0.712–0.763)	0.661 (0.627–0.699)	0.332 (0.315–0.350)	0.123 (0.094–0.153)
Longformer	0.773 (0.751–0.797)	0.745 (0.722–0.772)	0.278 (0.256–0.300)	0.273 (0.235–0.318)
BioMed RoBERTa	0.806 (0.785–0.826)	0.768 (0.745–0.791)	0.259 (0.241–0.279)	0.265 (0.224–0.306)
Bio+ClinicalBERT	0.829 (0.810–0.849)	0.795 (0.774–0.815)	0.252 (0.229–0.274)	0.330 (0.282–0.376)
GAT-CN	0.850 (0.833–0.867)	0.812 (0.791–0.831)	0.236 (0.218–0.253)	0.311 (0.271–0.353)

*AUROC* area under receiver operating characteristic curve, *AUPRC* area under precision-recall curve, *HL* Hamming loss, *SA* subset accuracy, *CI* confidence interval.

In the multi-label settings, GAT-CN achieved an HL of 0.236 (95% CI: 0.218–0.253), meaning that on average 23.6% of all possible labels were predicted incorrectly. The model achieved an SA of 0.311 (95% CI: 0.271–0.353), indicating that 31% of the patient notes had all diagnosis categories correctly identified. As HL penalizes each individual label error and SA requires all labels to be correct, these results reflect the model’s overall consistency across multiple symptom classes.

### Diagnosis labels from EHR, annotation, and model prediction

**Fig. 2:**
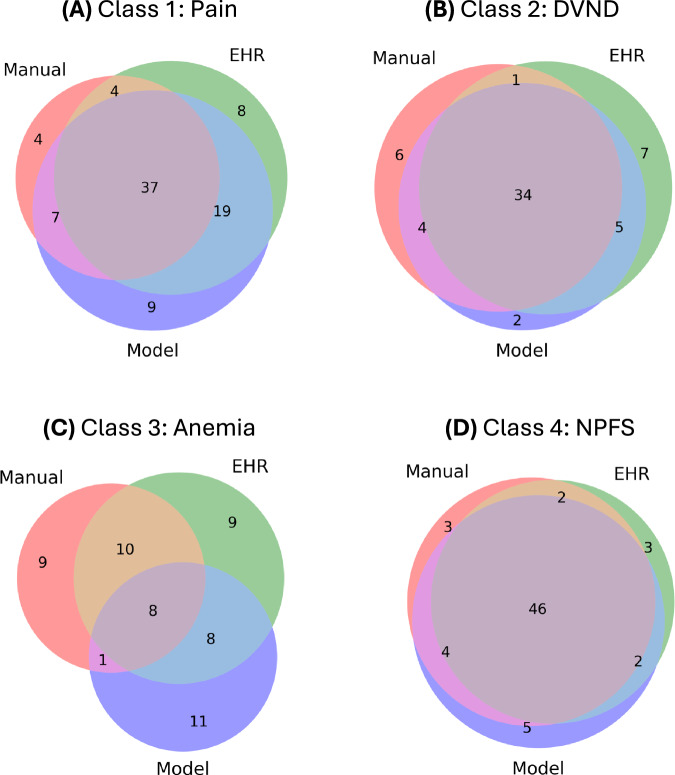
Overlap of diagnosis labels from EHR, manual annotations, and model predictions. This figure presents Venn diagrams comparing diagnosis labels from structured Electronic Health Records (EHR), manual annotations (Manual), and GAT-CN predictions (Model) for four post-chemotherapy diagnosis categories. Each diagram shows the number of patients identified by one, two, or all three sources, with overlapping regions indicating shared diagnoses and non-overlapping areas highlighting discrepancies. **A** Overlap between EHR labels, manual annotations, and model predictions for pain-related diagnoses. **B** Overlap between EHR labels, manual annotations, and model predictions for gastrointestinal-related diagnoses, including diarrhea, vomiting, nausea, dehydration (DVND). **C** Overlap between EHR labels, manual annotations, and model predictions for anemia. **D** Overlap between EHR labels, manual annotations, and model predictions for immune-related complications, including neutrophilia, pneumonia, fever, sepsis (NPFS).

Figure 2 depicts the overlap of diagnosis labels from EHR, manual annotations, and model predictions via a Venn diagram for each symptom diagnosis class in our test set. Substantial overlap is observed between DVND and NPFS, whereas Anemia shows minimal overlap compared to the other classes. Manual annotations aligned more closely with model predictions than with structured EHR labels, particularly for Pain, DVND, and NPFS. Additionally, both the annotators and the model identified diagnoses for patients that were absent from the structured EHR. The annotators revealed 11 additional patients for Pain (10.4%), of which GAT-CN correctly identified 7 (63.6%); 10 with DVND (9.4%), of which 4 (40%) were identified; 10 with Anemia (9.4%), of which 1 (10%) was identified; and 7 with NPFS (6.6%), of which 4 (57.1%) were identified.

### Model calibration and clinical utility

**Fig. 3:**
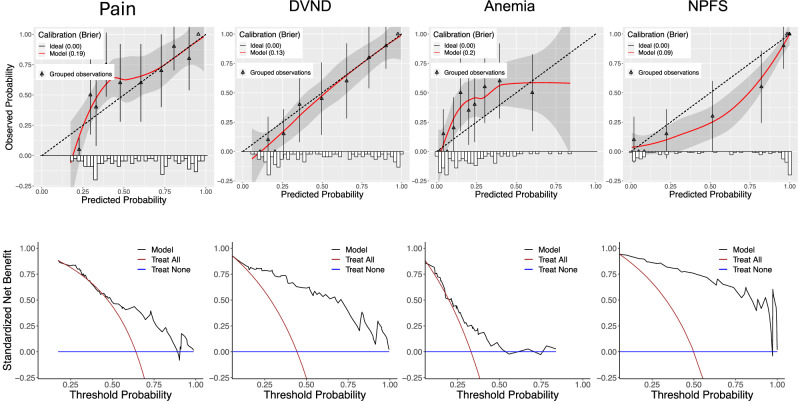
Model calibration and decision curve analysis for post-chemotherapy diagnosis classes. This figure presents the calibration and standardized net benefit analysis of the GAT-CN for predicting four post-chemotherapy diagnosis categories in the test set. Top row: Calibration plots compare predicted probabilities with observed outcome rates for each diagnosis class. Predictions were grouped into ten equal-sized probability bins. The dashed diagonal line indicates perfect calibration, while the solid red line shows model calibration. Gray shading represents 95% confidence intervals for observed outcome rates within each bin, calculated using the normal approximation. Histogram bars at the bottom show the distribution of predicted probabilities. Bottom row: Decision curve analysis shows the standardized net benefit across decision thresholds based on the predicted probability of diagnosis. The black line represents the model, while the red and blue lines represent treat-all and treat-none strategies, respectively. Each observation corresponds to an individual patient in the test set represented by a single aggregated clinical note (Pain: *n* = 68; DVND: *n* = 47; Anemia: *n* = 35; NPFS: *n* = 53).

Model calibration varied across outcome classes. GAT-CN demonstrated the best calibration for the DVND and NPFS classes, with low Brier scores of 0.13 and 0.09, respectively, and showed high net benefit compared with treat-all strategies (Fig. 3). In contrast, performance was lower for pain and anemia classes. For these outcomes, the model underestimated the prediction probabilities, and clinical utility was limited to a narrow range of decision thresholds relative to the treat-all strategy.

### Subgroup performance analysis

Table 3 shows the sample-averaged F1 scores (a multi-label performance metric) of GAT-CN across subgroups, including gender, race, ethnicity, cancer group, cancer stage, insurance coverage, ACU event type, number of diagnosis categories, and number of notes. The model showed a significant performance disparity on ethnicity (Mann–Whitney test, *p* = 0.02) and class counts (Kruskal–Wallis test, *p* = 0.04). However, post-hoc pairwise comparisons using Dunn’s test did not reveal significant pairwise differences between class count categories. In contrast, the model demonstrated performance parity across cancer types and stages.

**Table 3:** Subgroup GAT-CN model performance stratified by demographic and clinical categories using sample-averaged F1 scores in the multi-label setting

	F1-score	Test statistic	*P* value
Overall	0.68		
Sex		1686.5	0.07
Female	0.73		
Male	0.61		
Race		5.5	0.14
White	0.63		
Asian	0.71		
Other	0.79		
Black	1.00		
Ethnic group		7.75	**0.02**
Non-Hispanic	0.64		
Hispanic	0.82		
Other	1.00		
Cancer group		6.33	0.7
Hematopoietic lymph	0.70		
Genitourinary	0.68		
Pancreas	0.73		
Breast	0.76		
Lung thoracic	0.65		
Head/Neck	0.46		
Gastrointestinal	0.64		
Gynecologic	0.54		
Sarcoma	0.83		
Skin	0.99		
Cancer stage		1.48	0.69
Stage 0–I	0.53		
Stage II	0.72		
Stage III	0.70		
Stage IV	0.61		
Insurance		2.46	0.29
Medicare	0.61		
Private	0.74		
Medicaid	1.00		
ACU event		1077.5	0.69
Emergency services	0.68		
Hospitalization	0.67		
Class count		6.47	**0.04**
1 class	0.54		
2 classes	0.74		
>2 classes	0.79		
Note count		0.39	0.82
1 note	0.68		
2 notes	0.67		
> 2 notes	0.69		

The table presents statistical comparisons of GAT-CN performance across patient subgroups, including sex, race, ethnicity, cancer group, cancer stage, insurance coverage, ACU event type, number of diagnosis categories, and number of notes. Performance is evaluated using the sample-averaged F1-score on the manually annotated test set. For subgroups with two categories, differences were assessed using two-sided Mann–Whitney *U* tests. For subgroups with more than two categories, differences were assessed using two-sided Kruskal–Wallis tests. Test statistics (*U* or *H*) and exact *P* values are reported. Statistical significance was assessed at *α* = 0.05, and *P* values below 0.05 are indicated in bold. No adjustments for multiple comparisons were applied.

## Discussion

In this study, we introduce a graph-augmented transformer model for extracting post-chemotherapy symptoms that are temporally associated with cancer therapy-related toxicities, emphasizing the importance of patient-centered monitoring and management. Timely detection of these symptoms is critical for reducing ACU events and improving patient care. Our model, GAT-CN, is specifically designed to capture symptom mentions following chemotherapy initiation, temporally aligned with ACU events within 30 days. By integrating Bio+ClinicalBERT^18^ with a heterogeneous graph framework trained via GraphSAGE^19^, GAT-CN leverages the rich, complex information embedded in clinical notes. This architecture integrates local linguistic context with broader corpus-level patterns, enabling the model to learn not only from how symptoms are described but also from their relationships across patients. GAT-CN achieves strong performance in detecting OP-35 symptom diagnoses associated with ACU^11^ and outperforms existing transformer-based approaches such as BioMed RoBERTa^20^ and Longformer^21^. The utility of GAT-CN lies in its ability to transform unstructured clinical notes, often complex, noisy, and voluminous, into actionable insights that support timely interventions^22^. By linking domain knowledge through a clinically validated diagnosis vocabulary—consistent with prior studies^23^—and applying GNNs to connect clinical notes with diagnosis-related terms, our approach exemplifies how advanced digital tools enhance the monitoring and management of chemotherapy-related symptoms.

These improvements are particularly notable given the limitations of existing methods for symptom extraction. Previous approaches for symptom extraction, including rule-based and transformer-based models, often struggle to capture nuanced clinical associations, particularly in settings with limited annotated data^24^. While transformer models rely on sequence-level patterns, they typically require large datasets to effectively learn contextual associations. Graph-based methods such as TextGCN^25^ and MedText^26^ have demonstrated the value of leveraging graph structures for textual and biomedical data, but these approaches depend on transductive GCNs, which require a fixed static graph and do not generalize well to unseen data. Although GraphSAGE is trained on a graph constructed from the training data, it learns inductive aggregation functions that generalize to unseen notes based on their features and local graph structure, without requiring retraining or full graph reconstruction. This inductive capability enables more robust symptom extraction for the multi-label task of OP-35 post-chemotherapy symptoms, even with relatively small, annotated datasets.

IAA varied markedly across symptom classes, reflecting the inherent challenges in identifying symptoms from clinical notes. Agreement was highest for symptoms anchored by objective cues (e.g., fever, lab abnormalities) and lower for subjectively described or implicitly documented symptoms (e.g., pain, anemia)^27,28^. This variability also illustrates why simple rule-based methods struggle, as they tend to capture many mentions but conflate nuance, yielding high sensitivity with poor precision^24,29^. In contrast, GAT-CN leverages narrative context and relationships among clinical symptoms, enabling more precise and stable extractions and better handling of co-occurring symptoms within the same note. Taken together, these findings emphasize both the complexity of symptom representation in real-world documentation and the value of graph-augmented, context-aware approaches over rule-based methods.

In the multi-label setting, the model achieved an HL of 0.236 and an SA of 0.311, indicating that despite some label errors, GAT-CN effectively captured multiple co-occurring symptoms jointly—a task inherently more difficult than single-label prediction. Error analysis revealed that misclassifications primarily stemmed from ambiguous or implicit symptom mentions, overlapping symptom categories, and sparse documentation of certain symptoms such as anemia. In the graph component, highly connected or sparsely linked nodes occasionally propagated irrelevant information, leading to confusion across co-occurring symptom classes. These factors underscore the inherent complexity of multi-label symptom extraction from clinical text and point to areas for refining contextual and graph representations using the graph-attention mechanism^30^ in future work.

Our approach addresses the longstanding challenge of incomplete and fragmented EHRs, where structured data often omit key clinical details needed to understand patients’ conditions in cancer care^31^. In this study, manual annotations were more consistent with model predictions than with structured EHR labels, particularly for pain, gastrointestinal symptoms (DVND), and infection-related diagnoses (NPFS). This finding demonstrates the model’s ability to extract clinically relevant symptoms directly from unstructured clinical notes, achieving accuracy comparable to human annotations. Among the four diagnosis classes, performance was the lowest for the anemia class. This limitation likely reflects several factors: the small number of anemia cases in the dataset, lower IAA between EHR and manual labeling, and the reliance on structured lab values, rather than narrative descriptions, for anemia diagnosis^32–34^. Moreover, anemia terms were underrepresented in the diagnosis vocabulary, making it more difficult to learn accurate graph-based representations. To improve annotation reliability and model performance for such cases, future work should include clearer annotation guidelines, calibration among annotators, and consensus adjudication of disagreements^28^.

By leveraging natural language processing (NLP) and graph-based approaches to enrich structured EHR data with vital information extracted from clinical notes^35^, we can refine symptom capture and enhance the management of toxicity through improved monitoring and patient-specific interventions^12,13^ For example, if a patient’s past notes suggest recurrent nausea or prior infection-related ACU, this information could alert clinicians to proactively adjust antiemetic or antimicrobial strategies in future treatment cycles. Similarly, documented patterns of fatigue or fever could guide tailored monitoring or earlier supportive care. This integration emphasizes the transformative potential of digital tools to make cancer care more effective and responsive to patient needs. Building on these insights, we outline below how they can inform complication prevention and support tailored interventions throughout the care continuum.

Our findings highlight the important role of advanced digital tools like GAT-CN in automating the extraction of clinically relevant information from unstructured data to support clinical decision-making and improve patient outcomes in oncology. Building on existing literature^12,13^, this study captures valuable clinical details documented in notes but not reflected in structured data. By effectively extracting and analyzing these unstructured notes across the longitudinal patient trajectory, our model empowers healthcare providers to identify patients at higher risk for chemotherapy-related toxicities^36^. This enables timely interventions and supports the development of patient-centered care plans, improving the overall management of chemotherapy. Moreover, the integration of these insights into clinical workflows can help oncologists proactively monitor patient symptoms, potentially reducing ED visits and hospitalizations.

Additionally, our model’s ability to identify patient diagnoses from text has practical implications for early symptom detection. By analyzing patient communications, such as emails following chemotherapy^37^, the model can detect early signs of post-chemotherapy side effects^38^. This capability empowers healthcare providers to promptly recognize and address potential health issues, leading to improved patient outcomes and overall enhancements in healthcare delivery^39^. Targeted early interventions may also help prevent ACU episodes after chemotherapy, thereby reducing financial burdens and alleviating the suffering experienced by cancer patients. In this way, it supports a paradigm shift in leveraging unstructured data for real-time clinical decision-making, empowering healthcare providers to transition from reactive to preventive care models.

The success of initiatives like GAT-CN, which combine advanced computational techniques with clinical expertise, depends on fostering interdisciplinary collaboration among data scientists, clinicians, and healthcare administrators^40,41^ By bridging the gap between technology and clinical practice, we can ensure that innovations are tailored to address the complexities and nuances of patient care in oncology^42^. This collaborative approach is essential for driving the adoption of new methodologies and ensuring that they translate into practical solutions that optimize patient safety and reduce the burden of chemotherapy-related toxicities. Ongoing investment in these collaborations will be the key to advancing cancer care and developing cutting-edge tools that prioritize patient well-being^43^.

While our study demonstrates meaningful advancements in identifying post-chemotherapy symptoms from clinical notes, several limitations should be acknowledged. First, the study was conducted at a single cancer center with a single holdout test set, limiting generalizability. Nonetheless, the successful use of Bio+ClinicalBERT^18^, pretrained on notes from a Boston-based health system, suggests broader applicability^44^. Future work should validate GAT-CN on external multi-institutions. Second, variability in clinical language may result in inconsistencies in symptom documentation, potentially leading to misinterpretations of patient outcomes^45,46^ However, our graph-based framework mitigates this by linking related symptoms to validated diagnosis classes using embedding vectors, which reduces the impact of lexical variation. Moreover, because GAT-CN employs GraphSAGE, it enables inductive embeddings for unseen notes, potentially enhancing generalizability across institutions, though rare toxicities may still be underrepresented. Third, we segmented notes into ≤512-token chunks to align with standard transformer baselines (e.g., Bio+ClinicalBERT, BioMed RoBERTa), ensuring fair comparison but limiting potential benefits from long-context models such as Clinical-Longformer. As a model-agnostic framework, GAT-CN can readily incorporate these architectures in future work to capture longer contextual dependencies in clinical text. Fourth, we focus exclusively on symptom extraction from post-chemotherapy notes. While the model was trained on clinical notes temporally associated with a 30-day ACU period, when treatment toxicities often emerge, GAT-CN detects/classifies symptoms but does not quantify therapy-toxicity associations, which may be implicit in text. Fifth, reliance on unstructured text may lead to missing information when documentation is sparse, poorly articulated, or mostly based on structured data^47^. Future work should integrate structured and unstructured data for a more comprehensive view of patient health^48^. Finally, subgroup performance disparities may arise, particularly across ethnic groups, due to underrepresentation in training data or differences in documentation practices. Future work should broaden cohort diversity, monitor subgroup-level performance, and adopt fairness-aware or hybrid approaches integrating structured and unstructured data to mitigate documentation- and data-related biases. While these limitations frame important directions for future work, they do not detract from the contributions and potential impact of GAT-CN in enhancing post-chemotherapy symptom monitoring.

In conclusion, our study demonstrates the considerable promise of leveraging advanced NLP and GNN to improve the detection of post-chemotherapy toxicity symptoms. By effectively extracting clinically relevant information from unstructured clinical notes, GAT-CN enables more accurate symptom detection, providing actionable insights that could inform future patient symptom monitoring and supportive care strategies. These advancements contribute to a more precise understanding of patient experiences following chemotherapy and support the development of patient-centered oncology care. Looking ahead, the integration of such digital tools has the potential to enhance early symptom detection and guide interventions, thereby reducing unnecessary acute care use and improving health outcomes for cancer patients. Continued collaboration between data scientists and clinicians will be essential for translating these innovations into effective and patient-centered clinical workflows.

## Methods

This study complies with all relevant ethical regulations and was approved by the Stanford University Institutional Review Board (IRB protocol 47644), which granted a waiver of informed consent. We conducted a methodology development and a validation study using retrospective EHR data. The study followed the Transparent Reporting of a Multivariable Prediction Model for Individual Prognosis or Diagnosis^49^ (TRIPOD) and Minimum Information for Medical AI Reporting^50^ (MINIMAR) guidelines.

### Cohort selection

**Fig. 4:**
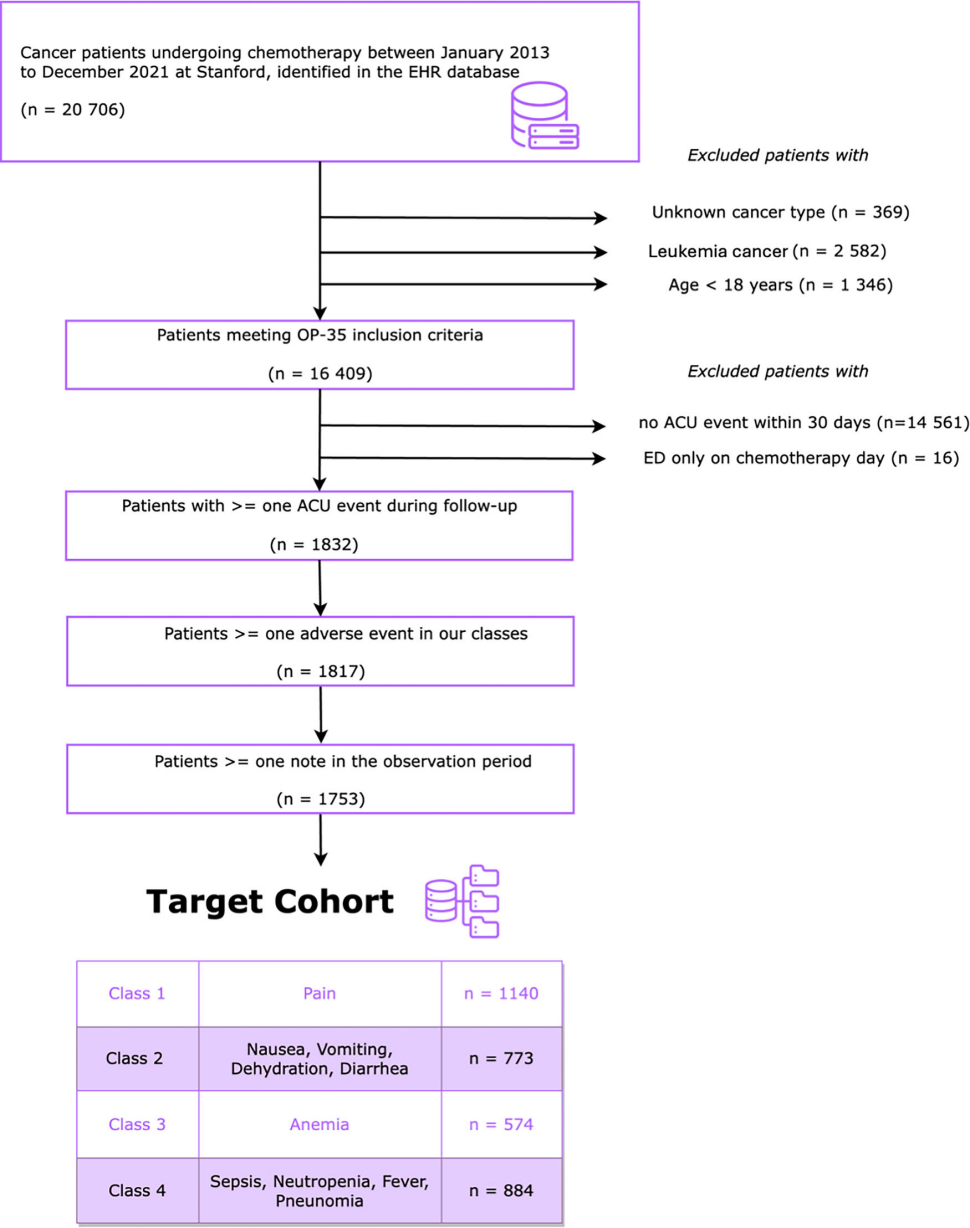
Phenotyping rules for target cohort extraction. This figure illustrates the selection process for cancer patients undergoing chemotherapy at Stanford (2013–2021). After applying OP-35 inclusion criteria, patients with unknown cancer types, leukemia cancers, or those under 18 were excluded. Only those with at least one emergency department (ED) visit or hospitalization within 30 days of chemotherapy initiation were included, excluding those with an ED visit only on the day of chemotherapy. Patients diagnosed with OP-35 post-chemotherapy diagnoses and with available text records during follow-up formed the final target cohort.

**Fig. 5:**
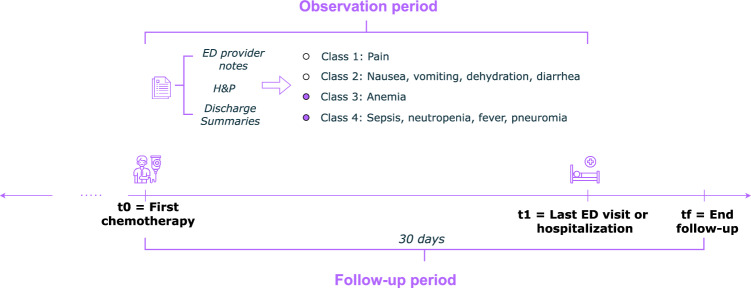
Problem definition. The index date (${t}_{0}$) marks the start of chemotherapy, defined as the first injection of the initial cycle. The intervention date (${t}_{1}$) represents the last emergency department (ED) visit or hospitalization within the 30-day follow-up period (${t}_{f}-{t}_{0}$). During the observation period (${t}_{1}-{t}_{0}$), patient notes—including ED provider notes for ED patients, History and Physical (H&P) notes, and Discharge Summaries for inpatient patients—are collected. The model categorizes patients into four predefined post-chemotherapy diagnosis classes.

Eligible patients had a cancer diagnosis defined by ICD codes and received chemotherapy using CPT codes as outpatients at Stanford Health Care (SHC) between January 2013 and December 2021^12^. Oral chemotherapy was excluded from the study due to difficulties in identifying its administration and link to ACU events. Following the criteria outlined by the CMS 2023 Measure Updates and Specifications Report, Hospital Outpatient Quality Reporting Program^51^, we excluded patients with unknown cancer types, patients with leukemia, and those under 18 years of age. Other hematologic malignancies, including lymphoma and myeloma, were retained in the cohort. From the remaining cohort, we included patients who were hospitalized or visited the ED within 30 days of their first chemotherapy session, excluding ED visits that occurred on day 0, i.e., the day on which chemotherapy was administered. For multiple ACU events within 30 days of chemotherapy, all notes until the last event were considered. Patients who were diagnosed with at least one OP-35 post-chemotherapy diagnosis—pain, nausea, vomiting, dehydration, diarrhea, anemia, neutropenia, fever, sepsis, and pneumonia—during their ACU episodes were included, as long as they had at least one clinical note documented up to the last ACU event within their 30-day period (Figs. 4 and 5). Notes were filtered for post-chemotherapy documentation to focus on therapy-related symptom detection. Demographic and clinical characteristics at the time of index diagnosis were abstracted from the structured EHR, including age, sex, race and ethnicity, cancer type and stage, and patient insurance type (private, Medicare, or Medicaid). Sex was obtained from the EHR demographic field recorded during clinical registration. Because the goal of this study was to evaluate the performance of an NLP model rather than investigate biological differences, sex was not used as a primary modeling variable but was considered in subgroup analyses to assess potential differences in model performance.

### Study outcome

The primary study outcome was the OP-35 diagnoses corresponding to each patient’s post-chemotherapy symptoms over the course of their observation^52^. The OP-35 measure defines ten post-chemotherapy diagnoses: pain, nausea, vomiting, dehydration, diarrhea, anemia, neutropenia, fever, sepsis, and pneumonia. For the purposes of this study, these ten diagnoses were grouped into four clinically actionable categories to simplify model training and evaluation: (Class 1) Pain; (Class 2) Diarrhea, Vomiting, Nausea, Dehydration (DVND); (Class 3) Anemia; and (Class 4) Neutrophilia, Pneumonia, Fever, Sepsis (NPFS). The diagnoses within each class share overlapping symptom presentations, clinical management strategies, and related comorbidities, providing the clinical rationale for their grouping in this study. For each patient, a four-element binary vector was used as a label. Each element in the vector represented one of the four classes, with a value of 1 indicating that the patient had at least one diagnosis related to that class. In contrast, a value of 0 was assigned if the patient did not have a relevant diagnosis for that class. Since the study focused exclusively on positive patients, each patient in the study population was assigned to at least one diagnostic class, resulting in a unit vector, and could belong to up to four classes, forming an all-ones vector.

### Diagnosis vocabulary and note preprocessing

We created the OP-35 Diagnosis Vocabulary (ODV), a curated symptom lexicon tailored to the four OP-35 post-chemotherapy diagnostic categories, noted above. Each category was assigned a distinct set of terms derived from the expertise of oncologists and emergency physicians, supplemented with SNOMED CT concepts, common abbreviations (e.g., N&V for nausea and vomiting), and synonyms from UMLS and the literature. The lists were refined by removing terms absent from the training notes or judged irrelevant by clinicians, while ensuring that each term was specific to a single diagnostic class. The ODV was then used to identify relevant clinical notes for each patient and to generate diagnosis term nodes in our contextual graph representation. To address the inherent variability in clinical documentation, we incorporated common abbreviations and synonymous variants into the symptom vocabulary and leveraged contextualized embeddings, rather than strict string matching, to support robust symptom identification.

Given the extensive length and diverse content of clinical notes, we used regular expressions to extract sentences in the notes that mentioned at least one vocabulary term, along with their two neighboring sentences above and below to preserve contextual information. Consequently, each note was condensed into a compilation of distinct sentences, which were then aggregated in chronological order to create patient-level notes. Subsequent preprocessing steps included the removal of URLs and unusual characters, as well as the reduction of noise by eliminating similar sentences. The output of these steps yielded preprocessed patient-level notes ready for input to the classification model for training and evaluation.

### Model development

We introduce a symptom diagnostic model, GAT-CN, which integrates Bio+ClinicalBERT^18^, with GraphSAGE^19^, an inductive GNN, to detect post-chemotherapy symptoms from clinical notes (Fig. 1). In this architecture, Bio+ClinicalBERT provides the initial embeddings for patient-note nodes, while GraphSAGE refines these embeddings within a heterogeneous graph that encodes relationships between notes and diagnosis terms. This design allows GAT-CN to capture both local language context and higher-order associations between clinical concepts. The heterogeneous graph contained two node types: patient clinical note nodes and diagnosis term nodes (generated from ODV), and two edge types: unweighted edges connecting notes to their associated diagnosis terms, and weighted edges connecting terms that co-occurred in a note, where edge weights reflected positive pointwise mutual information.

Patient notes are encoded with Bio+ClinicalBERT^18^; for long notes, the text is chunked and mean-pooled to yield a single vector ${{{{\bf{x}}}}}_{v}$, which serves as the initial embedding for the corresponding patient-note node (${h}_{v}^{0}=\,{{{{\bf{x}}}}}_{v}$). In the GraphSAGE^19^ framework, node representations are updated through message passing, where each node aggregates information from its neighbors across depths $k$.

The first layer $\left(k=1\right)$ updates the initial patient-note embedding by integrating the aggregated representations of its immediate neighbor diagnosis-term nodes. Each diagnosis-term nodes are represented by BioWordVec^53^ embeddings of dimension $d$, concatenated with a single scalar encoding the diagnostic class label (an integer between 0 and 3), yielding a $(d+1)$-dimensional vector for each term. Neighbor embeddings are processed through a convolutional layer, followed by 1D max-pooling and a fully connected (FC) layer to obtain the aggregated neighbor representation ${h}_{{{{\mathcal{N}}}}(v)}^{(1)}$. The updated patient-note embedding is computed via a ReLU-activated FC layer (see Fig. 1 for an overview of the GAT-CN architecture at depth $k=1$). For higher depths ($k > 1$), the same process is recursively applied as Equation 1:

1 $${h}_{v}^{k}={ReLU}\left({concat}\left({h}_{v}^{k-1},\,{h}_{{{{\mathcal{N}}}}\left(v\right)}^{k}\right){\left({W}^{k}\right)}^{T}+{b}^{k}\right)$$

where ${W}^{(k)}$ and ${{{{\bf{b}}}}}^{(k)}$ denote the learnable weight matrix and bias vector. After $k$ hops, the GraphSAGE-updated patient-note embedding ${h}_{v}^{k}$—initialized from Bio+ClinicalBERT but not concatenated or residually combined with it—is passed to a linear multi-label head with four sigmoid outputs, yielding the probabilities for each diagnostic class. Only clinical note nodes are used for prediction.

GAT-CN was trained jointly in an end-to-end manner. Bio+ClinicalBERT was initialized with publicly available pretrained weights and fine-tuned during training, while the GraphSAGE component was randomly initialized and trained simultaneously. Model optimization was performed exclusively on the training split, with validation used for early stopping and hyperparameter tuning, and the test set held out entirely for final evaluation.

Model training was performed over 10 epochs with early stopping if the validation loss did not decrease for five consecutive epochs. We used the AdamW optimizer^54^ with a learning rate of 3e-5 (reduced by a factor of 10 after two stagnant epochs), binary cross-entropy loss, and a 20% dropout rate. Batch size was set to 32, and weight decay was 0.01. Model parameters were initialized using Xavier uniform initialization^55^. For GraphSAGE, we used a maximal search depth of 2, sampling 150 term-node neighbors and 50 note-node neighbors per layer. The aggregation function applied a CNN kernel of sizes^1–3^, each with 50 output channels. Approximately 6% of the cohort was held out for testing, with 20% of the remaining data used for validation. The final model was selected based on the epoch with the lowest validation loss and evaluated on the test set to ensure optimal generalization.

### Model evaluation

To evaluate model performance, clinical notes documented prior to ACU were manually annotated to establish ground truth for symptom presence, rather than to predict subsequent ACU. Four co-authors (MS, MP, BTR, DWB), with differing levels of clinical expertise, manually annotated the test set to determine whether a patient’s clinical notes contained documentation of the OP-35 symptoms. Inter-annotator agreement was assessed on six patients using Krippendorff’s alpha^56^. Final labels were determined using a weighted voting scheme that reflected annotators’ clinical expertise, with weights of 0.40 assigned to the oncologist (DWB), 0.30 to the medical doctor (BTR), and 0.15 each to the remaining two annotators (MS and MP). This weighted voting approach determined the final labels for these patients. The remaining test patients were split equally among annotators. Annotations were compared with EHR labels and model predictions; agreement was quantified with Krippendorff’s alpha, and Venn diagrams visualized overlaps across sources.

Symptom extraction was evaluated at the patient-note level. A symptom was considered correctly identified if the model detected at least one mention corresponding to the annotated symptom (flexible match), rather than requiring exact span matching, to account for variability in clinical documentation. Model performance was assessed per symptom class using receiver operating characteristic (ROC) and precision-recall (PR) curves. We reported weighted averages of the area under ROC (AUROC) curve and the area under PR curve (AUPRC) to summarize performance across all prediction thresholds. In addition, we reported weighted averages of precision, recall, and F1-score at a decision threshold of 0.5. The F1-score, the harmonic mean of precision and recall, is particularly informative in clinical settings as it balances sensitivity to true positives with minimization of false alarms.

To evaluate performance in the multi-label setting, where a single patient’s notes may contain multiple correct labels, we used three complementary metrics: (1) Hamming loss (HL): the fraction of incorrectly predicted labels out of the total number of labels across all samples; (2) Subset accuracy (SA): the strictest measure, reflecting the percentage of notes for which the entire predicted label set exactly matches the true set; and (3) Sample-averaged F1-score: the F1-score computed for each note and then averaged across all notes.

We used the sample-averaged F1-score for subgroup analysis to assess performance variations across patient characteristics and to capture the model’s ability to identify all relevant symptom classes per note, reflecting joint multi-label performance rather than independent class-based metrics like AUROC. This metric provides a balanced summary of precision and recall, offering more interpretable results than stricter measures such as HL or SA. To evaluate clinical utility, we conducted decision curve analysis using standardized net benefit^57^ across a range of clinically relevant decision thresholds. Calibration was assessed for each of four OP-35 outcome classes using calibration plots with grouped observations and Brier scores. To quantify uncertainty, we applied bootstrap resampling with 1000 iterations to estimate 95% confidence intervals (CIs) for model performance.

To contextualize the performance of GAT-CN, we benchmarked it against three transformer-based models—Longformer^21^, BioMed RoBERTa ^20,58^, and Bio+ClinicalBERT^18^—trained with default parameters. (see Supplementary Section Hyperparameter Selection and Training Dynamics for details) Additionally, we included two baseline approaches to illustrate the performance gap between traditional and advanced transformer-based models. First, a random forest classifier trained on TF-IDF features served as a representative rule-based ensemble method, where each decision tree applies a series of if–then rules and the ensemble aggregates these rules to improve robustness. For this random forest baseline, hyperparameters were tuned on the training set using validation-based model selection. Second, a simple, flexible string-matching approach labeled notes based on the presence of at least one term from a curated symptom dictionary; for example, a note containing any pain-related term was classified as pain-related. This rule-based baseline highlights the challenges of symptom extraction from unstructured text and shows the need for more advanced, contextual, graph-based approaches such as GAT-CN.

### Computational resources

Model training and evaluation were performed primarily on a machine with a 10-core CPU (2.4 GHz) and 32 GB of RAM, where the Bio+ClinicalBERT and GraphSAGE components were trained jointly. Depending on hyperparameter settings, average run times per epoch ranged between 1 and 2 h, with early stopping typically reached after 6–8 epochs. As a proof-of-concept, we also tested training on an Apple M1 GPU, where per-epoch runtime was substantially reduced (~9 min per epoch), illustrating that modern GPUs can dramatically accelerate training. These observations indicate that GAT-CN can be trained efficiently on standard CPU hardware and that GPU acceleration can facilitate larger-scale validation across datasets.

### Statistical analysis

A statistical analysis was conducted to investigate variations in the model’s performance across different patient subgroups. Subgroups were defined based on patient characteristics, including race, ethnicity, cancer group, and stage, as well as type of ACU event, number of diagnosis categories, and number of notes. For each subgroup, we calculated the sample-averaged F1-score. To assess significant performance differences across subgroups, non-parametric tests were used because they do not assume normality of the data distribution. Specifically, we used the Mann–Whitney *U* test for comparisons between two categories and the Kruskal–Wallis test for analyses involving more than two categories. The significance level was set at *α* = 0.05, and all tests were two-tailed. When the Kruskal–Wallis test indicated significant differences, Dunn’s post-hoc test with Bonferroni correction was applied to identify specific subcategories with divergent model performance.

## Supplementary information

Supplementary Information

Transparent Peer Review file

## Data Availability

The dataset used in this study contains protected health information derived from electronic health records and, therefore, cannot be made publicly available due to institutional and ethical restrictions. Access to de-identified data may be granted to qualified researchers subject to approval by the Stanford University Institutional Review Board and completion of appropriate data use agreements. Requests for access should be directed to the corresponding author and will be reviewed to ensure compliance with institutional policies and patient privacy regulations. Decisions regarding access are typically provided within 4 weeks of request. Approved researchers will be granted access for the duration specified in the data use agreement.

## Code availability

The source code and curated dictionary developed for this study are publicly available at https://github.com/su-boussard-lab/GAT-CN, enabling evaluation and use across diverse clinical datasets.
