# Supplementary material for: Graph augmented transformers improve chemotherapy toxicity symptom extraction from clinical notes
Source: Nat Commun. 2026 Apr 28;17:5829. doi: 10.1038/s41467-026-72347-2 (PMC13332229; doi:10.1038/s41467-026-72347-2)
Supplement: Supplementary file 1 — Supplementary Information [file 41467_2026_72347_MOESM1_ESM.pdf]

Supplementary Materials

Hyperparameter Selection and Training Dynamics

We tuned hyperparameters empirically through a limited search on the validation set. Because GAT-CN is computationally intensive to train, performing k-fold cross-validation or exhaustive grid search was not feasible. Instead, we evaluated a small set of configurations for learning rate, dropout, batch size, and GraphSAGE neighbor sampling, selecting the final settings that consistently minimized validation loss while maintaining stable training behavior. Training and validation losses were monitored during all experiments, and convergence was stable across runs. For GraphSAGE, we tested different neighbor sampling ranges and kernel widths, choosing the configuration that balanced predictive performance with computational efficiency.

Transformer-based models, RoBERTa-base and Longformer-base, utilized pre-trained weights with architecture-specific configurations. Specifically, RoBERTa consisted of 12 transformer layers with a hidden size of 768 and 12 attention heads. The Longformer adopted the same base architecture but employed a sliding-window attention mechanism with a window size of 512.

Supplementary Table 1. Cancer Group Classification.

**Cancer Types**	**Seed Terms**
Head/Neck	["lip", "hypopharynx", "tongue", "oropharynx", "gum, floor of mouth and other mouth", "salivary gland", "nasopharynx", "larynx", "accessory, sinuses, middle and inner ear", "nasal cavity (including nasal cartilage)", "orbit and lacrimal gland, (excl. retina, eye, nos)", "thyroid gland"]
Lung Thoracic	["lung and bronchus", "mediastinum", "pleura"]
Genitourinary	["kidney", "renal pelvis, ureter", "adrenal glands", "urinary bladder", "other urinary organs", "testis", "penis and scrotum"]
Pancreas	["pancreas", "liver", "gallbladder and extrahepatic bile ducts", "intrahepatic bile ducts"]
Sarcoma	["retroperitoneum and peritoneum", "connective and soft tissue", "bones and joints"]
Gastrointestinal	["stomach", "small intestine", "appendix", "esophagus", "large intestine, (excl. appendix)", "rectum", "anal canal and anus"]
Gynecologic	["corpus uteri", "ovary", "cervix uteri", "other female genital", "vagina and labia"]
Hematopoietic Lymph	["blood, bone marrow and hematopoietic sys", "lymph nodes", "thymus", "spleen"]
Breast	["breast"]
Skin	["skin"]
This table presents the seed terms used to categorize each patient's cancer into one of the 10 cancer groups: Head/Neck; Lung Thoracic; Genitourinary; Pancreas; Sarcoma; Gastrointestinal; Gynecologic; Hematopoietic Lymph; Breast; Skin.	

Supplementary Table 2. Examples of OP-35 Vocabulary (ODV) terms in each diagnostic category.

**Diagnostic Class**	**# of Terms**	**Examples**
Pain	52	ach, tens, distress, tender, numb, burn, tingl, discomfort, cp, inflammation, ha, swollen, suffer, hurt, h/a, suffer
Diarrhea, Vomiting, Nausea Dehydration (DVND)	50	fluid, stool, abdom, gerd, emes, iron, fatigue, naus, vomit, bowel,fluid, gastro, electrolyte, n/v, bloody, reflux, abd, liquid, stomach, watery, cinv
Anemia	25	cbc, rbc, weak, hemoglobin, hematocrit, pale, cold, dizz, mucous, macrocytic, microcytic, lethargy, marrow, fatigue, tired
Neutropenia, Pneumonia, Fever, Sepsis (NPFS)	80	infect, cough, wbc, sob, bacteria, fever, infect, bp, cxr, bp, viral, virus, pcp, sbp, hypoxia, white blood cell, heart rate, hypertherm, shiver, f/c
This table showcases vocabulary examples for each post-chemotherapy diagnosis category. Pain comprises 52 terms, including ach (ache), tens (tension), h/a (headache). DVND consists of 50 terms, such as gerd (Gastroesophageal Reflux Disease). Anemia contained 25 terms, including CBC (Complete Blood Count) and rbc (Red Blood Count). NPFS encompassed 80 terms like wbc (White Blood Count), sob (shortness of breath).		

Supplementary Table 3. Pairwise inter-annotator agreement (IAA) analysis across diagnostic classes using Krippendorff's alpha.

Diagnoses		Nr. 1	Nr. 2	Nr. 3	Nr. 4	Average
Pain	Nr. 1	1.0	0.69	0.69	0.59	0.66
	Nr. 2	0.69	1.0	0.39	0.31	0.46
	Nr. 3	0.69	0.39	1.0	0.31	0.46
	Nr. 4	0.59	0.31	0.31	1.0	0.40
DVND	Nr. 1	1.0	0.59	1.0	0.06	0.55
	Nr. 2	0.59	1.0	0.59	-0.1	0.36
	Nr. 3	1.0	0.59	1.0	0.06	0.55
	Nr. 4	0.06	-0.1	0.06	1.0	0.007
Anemia	Nr. 1	1.0	0.59	1.0	0.06	0.55
	Nr. 2	0.59	1.0	0.59	-0.1	0.36
	Nr. 3	1.0	0.59	1.0	0.06	0.55
	Nr. 4	0.06	-0.1	0.06	1.0	0.007
NPFS	Nr. 1	1.0	1.0	1.0	1.0	1.0
	Nr. 2	1.0	1.0	1.0	1.0	1.0
	Nr. 3	1.0	1.0	1.0	1.0	1.0
	Nr. 4	1.0	1.0	1.0	1.0	1.0

This table presents the results of a pairwise inter-rater agreement (IAA) analysis on diagnostic labels for six test patients, employing Krippendorff's alpha. The analysis involves four annotators (Nr. 1, Nr. 2, Nr. 3, Nr. 4) with specialized medical expertise. Each cell reports the pairwise IAA between annotators, while the final column shows the average IAA for each annotator across all pairwise comparisons.

Supplementary Table 4. Comparison of rule-based, machine learning, and transformer-based models.

Model	Precision	Recall	F1-score
Rule-based string-matching	0.505	**0.995**	0.663
Random Forest	0.702	0.656	0.647
Bio+ClinicalBERT	0.750	0.744	0.743
GAT-CN	**0.780**	0.784	**0.780**
Performance is reported in terms of weighted average Precision, Recall and F1 score across four diagnostic classes. The best results are highlighted in bold.			

Supplementary Figure 1. ROC and PR curves for GAT-CN on post-chemotherapy diagnostic classes.

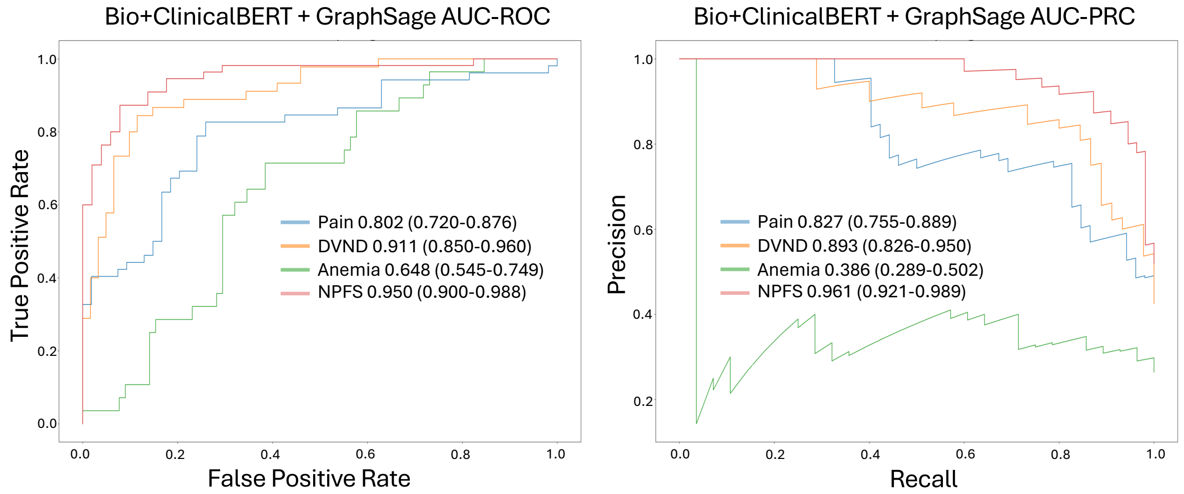

Receiver operating characteristic (ROC) and precision–recall (PR) curves of GAT-CN, a model combining Bio+ClinicalBERT and GraphSAGE, for four post-chemotherapy diagnostic classes evaluated on the manually labeled test set. DVND denotes diarrhea, vomiting, nausea, and dehydration, and NPFS denotes neutropenia, pneumonia, fever, and sepsis. Area under the curve (AUC) values are reported with 95% confidence intervals (CIs) in parentheses, estimated using 1,000 bootstrap iterations.
