# Supplementary material for: Graph augmented transformers improve chemotherapy toxicity symptom extraction from clinical notes
Source: Nat Commun. 2026 Apr 28;17:5829. doi: 10.1038/s41467-026-72347-2 (PMC13332229; doi:10.1038/s41467-026-72347-2)
Supplement: Supplementary file 2 — Transparent Peer Review file [file 41467_2026_72347_MOESM2_ESM.pdf]

nature portfolio

Peer Review File

Improving Symptom Extraction from Clinical Notes using a Graph Neural Network Approach for Enhanced Chemotherapy Adverse Event Monitoring

Corresponding Author: Professor Tina Hernandez-Boussard

This file contains all reviewer reports in order by version, followed by all author rebuttals in order by version.

Version 0:

Reviewer comments:

Reviewer #2

(Remarks to the Author)
Dr. Saquand and colleagues demonstrate an innovative approach to the use of NLP for extracting symptoms and adverse event monitoring in patients with cancer. Overall, this is an excellent, timely and novel article, and I appreciate the opportunity to review this important work. My comments are included below organized by section.

Introduction:
-The introduction is well written. It might be helpful to the reader to include rates of ACUs with cancer treatment and/or note the percentage of cancer spending on ACU to highlight the scope of the problem.
-In the introduction I think it would be helpful to explain a little more deeply what transformer-based architectures and graph neural networks are so that the reader better understands the connection of why the model is novel with the ability to uncover undocumented diagnoses.

Methods:
-I would not use the term validation when noting consultation with experts was used. I think it would be more appropriate to say refinement, as validation implies statistical testing, unless Kappa statistics or other validation statistics were compared with physician experts.
-The methods state that patients with “blood cancers” were excluded, but the results note that hematopoietic tumors were the most prevalent cancer type and discuss lymphoma patients. The authors should clarify the hematologic malignancies permitted in the study.

Discussion:
-I would also note that there is limited representation in the patient sample from certain patient races, and some cancer types are missing altogether (multiple myeloma, acute leukemia), which also can limit generalizability.
-The Discussion notes that this work builds on prior literature, but I think the Discussion would benefit from a little more direct elaboration on prior NLP work in this area and how this moves the field forward.

Tables and Figures:
-For Supplementary Table 1, I would suggest including a significantly larger number of example terms. This Table is very helpful for giving the reader of sense of what vocabulary is getting flagged.
-For Supplementary Table 2, the IAA values are fairly modest except for NPFS. Do the authors have a comment or explanation for the more modest IAA for pain and especially for DNVD and anemia?

(Remarks on code availability)
I apologize if I missed it but did not see the code in the files.

Reviewer #3

(Remarks to the Author)

The manuscript introduces a graph-based method, i.e., GAT-CN, designed to extract 36 chemotherapy-related symptoms from clinical notes. Evaluation demonstrates the method outperformed transformer-based models in multi-symptom labelling. However, there are some issues with the current manuscript.

Major concerns:

1. Graph-based text classification has been studies for years (e.g., the basic idea is to convert or create a graph for a given text, and then encode them with GNNs), yet the paper does not mention any of them as related work. Even for medical texts, there are many existing studies that adopt a similar idea. For example, https://dl.acm.org/doi/10.1145/3404835.3463062

2. The method itself is unclear with too many steps. It is very hard to follow. Figures illustrating the logistic connection between various steps could help to understand. For example, it is unclear how the "term nodes" are generated. The architecture of GAT-CN is also unclear, e.g., according to line 177 the BioClinicalBERT embeddings of text is used as node features, however in line 191 there seems a residual connection. Line 132-194 are confusing and it would be better to provide an overview figure of the architecture.

3. The experiments are insufficient and some details are missing. For example, it is unknown if GAT-CN has an advantage over other graph-based methods or even rule-based approaches. It would be interesting to see the results with the "term nodes" concatenated as a sequence rather than form a graph as the input. Some details are missing, including the number of nodes/edges for each input.

4. The study design was contradictory, they first claimed that "we excluded patients 104 with unknown cancer types, blood cancers, and those under 18 years of age." While in the result, they reported "Lymphoma tumor patients were more likely to be diagnosed with anemia 267 (n = 166, 28.9%)". As lymphoma is a type of blood cancer, the method is not convincing and rigorous.

5. The manuscript also claimed this is a "retrospective cohort study", which is a specific study method in traditional epidemiology. This is not accurate, the study is more like a methodology development.

6. The method focused on detecting cancer therapy-related toxicities, however, a large amount of pre-processing was done to select notes for study. The so-called novel method extracted symptoms only. The relation between cancer therapy and toxicities were established through the logic of "following chemotherapy initiation that are linked to ACU events 341 within 30 days." In this sense, it's not accurate to say the contribution of the method is for detecting cancer therapy-related toxicities. It is only symptom detection, which is similar to NER task in NLP.

7. The study collected a list of toxicity terms, however, the comparison to a rule-based method was not conducted. The study claimed that they "introduce a novel approach for detecting cancer therapy-related 337 toxicities". But only 4 classes of toxicities were focused on, the mention of 337 toxicities was an exaggeration. What are numbers of labels for each class?

8. It's not clear how the evaluation was done. Is it evaluated based on span match, exact match or flexible match? There is an inconsistency in evaluation metrics AUROC and F1 were used in various instances, why?

Error analysis is desired. For example:
what caused error in the results of IAA: "The overall IAA across the test set varied by class, with perfect agreement for NPFS (IAA = 1.0), fair agreement for Pain (0.59) and DNVD (0.49), and lower agreement for Anemia (0.26)." what caused errors in evaluation: "GAT-CN had a HL of 0.236 298 (95% CI 0.218–0.253), indicating that on average, approximately 23.6% of all labels 299 were predicted incorrectly." Reporting of results need more details, for example, what's the percentage----- "Specifically, they detected 7 patients with Pain, 1 with Anemia, 4 with symptoms 313 related to DVND, and 4 with diagnoses linked to NPFS." What's the impact of the percentage?

(Remarks on code availability)

Reviewer #4

(Remarks to the Author)
Summary
This paper proposed a natural language processing model to extract chemotherapy-related symptoms from clinical notes to integrate EHR information with unstructured clinical notes. This model demonstrates better performance in multi-symptom labelling compared to transformer models.
Comments:
1. The current evaluation relies primarily on a single holdout test dataset. The absence of additional external or diverse datasets might limit insights into the generalizability of the GAT-CN model across various clinical settings or institutions.
2. Currently, the model's performance analysis is primarily reported using the F1 metric. Including additional sensitivity analyses, such as precision-recall curves, ROC curves, or decision threshold analyses, would offer deeper insights and further contextualize the model's performance, particularly regarding its sensitivity and specificity trade-offs.
3. While the paper benchmarks the GAT-CN model against established transformer models, it should also include comparisons against newer state-of-the-art transformer models designed specifically for clinical text, such as Clinical-

Longformer/BigBird and BioClinical ModernBERT.
4. The supplementary materials lack detailed information regarding model training processes, specifically describing hyperparameter tuning procedures, parameter selection justifications, and including comprehensive training and evaluation loss curves.
5. In the methods section, the manuscript does not describe the computational resources of deploying this model, including detailed metrics about training time, hardware requirements, resource utilization, and scalability.
6. While the manuscript adequately addresses lower inter-annotator agreement scores for the Anemia classification, it lacks suggested strategies to enhance annotation reliability.
7. The discussion did not address subgroup performance disparities, particularly regarding ethnicity, offering detailed hypotheses for observed disparities and proposing concrete, actionable strategies to improve model fairness, such as diversifying training data, targeted fine-tuning, or fairness-aware algorithmic adjustments.
8. Code Availability is not provided or mentioned.
Minor comments:
Line 227-229, it is ambiguous to state that ‘we benchmarked it against four transformer models’, as only three transformer models were listed and the traditional Radom Forest classifier is not transformer model.

(Remarks on code availability)
Code availability is not mentioned/provided in the manuscript.

Reviewer #5

(Remarks to the Author)
I co-reviewed this manuscript with one of the reviewers who provided the listed reports. This is part of the Nature Communications initiative to facilitate training in peer review and to provide appropriate recognition for Early Career Researchers who co-review manuscripts.

(Remarks on code availability)

Version 1:

Reviewer comments:

Reviewer #2

(Remarks to the Author)
The authors have sufficiently addressed all of my prior comments. Thank you for the opportunity to review this work.

(Remarks on code availability)

Reviewer #4

(Remarks to the Author)
The authors have addressed previous comments. The author added more information about ACU and transformer/GAT-CN frameworks in the introduction section.
The paper now includes the single holdout test evaluation as a limitation in the discussion
The authors explain why they did not select more updated BERTs such as BioClinical ModernBERT for the study.
The authors include the computational information in the method section.
There are no other major or minor comments to this paper.

(Remarks on code availability)

Reviewer #5

(Remarks to the Author)
I co-reviewed this manuscript with one of the reviewers who provided the listed reports. This is part of the Nature Communications initiative to facilitate training in peer review and to provide appropriate recognition for Early Career Researchers who co-review manuscripts.

(Remarks on code availability)

Reviewer #6

(Remarks to the Author)

This manuscript describes an NLP GAT-CN model that is designed to extract symptoms temporally associated with chemotherapy-induced toxicities from clinical notes, towards predicting acute care utilization (ACU) events. In particular, GAT-CN embeds patient notes with the Bio+ClinicalBERT transformer encoder, which are then linked to diagnosis-related terms in a (pre)trained graph model. This was found to outperform transformer-only approaches.

The early prediction of ACU risk is an important and under-explored challenge. However, there may be a number of issues with the present form of the manuscript, in particular relating to the exact task definition for GAT-CN:

1. In the Results section, it is stated that the target cohort comprised 1753 patients who received acute care within 30 days of initiation of chemotherapy. However, in practical application for the proactive prediction of ACU events (as implied in the Introduction), the 30-day acute care status of patients would be necessarily unknown. If so, the applicability of the cohort to the intended task scenario might thus be further justified.

2. It is then stated that of the ACU patients (assumed to be the full cohort of 1753 patients in the target cohort), 95 experienced an ACU event during their observation period (defined as t_1 - t_0 in the problem definition in Figure 2, where t_f - t_0 = 30 days). It might be clarified as to whether the remaining (1753 - 95) patients had (one or more) ACU events after t_1 but before t_f (i.e. after the observation period), and whether the GAT-CN model is supposed to predict ACU risk only for this post-observation period (because the required EHR data is collected during the observation period).

3. In the Diagnosis Vocabulary and Note Annotation subsection, it is stated that the training and validation sets were labeled using patient OP-35 diagnosis codes in the EHR data, but the test set was manually annotated by four annotators. This difference in annotation between the training/validation and test sets might be explained - was annotation by diagnosis code inapplicable for the test set?

4. For the diagnostic-related terms, it might be clarified as to whether and how (possibly non-standard) abbreviations and misspellings were handled.

5. The specific data used to develop and validate the Bio+ClinicalBERT transformer encoder, as well as the GraphSAGE-trained graph model, might be clarified. The Model Development subsection stated that approximately 6% of the cohort was held out for testing, with 20% of the remaining data used for validation, but it is not fully clear as to whether the individual transformer and graph components of GAT-CN were trained/fine-tuned.

6. In the Discussion section, transductive GCNs were critiqued as requiring a fixed static graph that do not generalize to unseen data. However, the GraphSAGE framework appears to also yield a fixed static graph, for a given set of training data; it might be clarified as to whether this understanding is correct.

7. In the Model Evaluation subsection, it is stated that the ground truth for model performance evaluation was generated by manual annotation on patients' clinical notes (obtained before t_1, from Figure 2). However, if the intended objective is proactive prediction, the relevant ACUs would arise only after the notes were recorded (i.e. after t_1, before t_f). If so, it might be clarified as to whether the annotation using the clinical notes (obtained before t_1) would reflect future patient ACU (after t_1).

8. In the Model Evaluation subsection, it is stated that weighted voting was used to finalize labels. Details about how the relevant weights were assigned might be stated.

9. In the Model Evaluation subsection, benchmarking against existing transformer-based models and baseline approaches was described. An ablation experiment with different embeddings as input to the graph model might be appropriate, to support the chosen Bio+ClinicalBERT embedding.

10. For the Random Forest baseline approach, it might be clarified as to whether hyperparameter optimization had been attempted.

11. In Figure 3, it is stated that each diagnosis-term node combines a 200-dimensional BioWordVec embedding, with a 1-dimensional diagnosis-symptom class encoding. It might be commented as to whether the 1-dimensional encoding is sufficient, especially given the relatively low dimensionality of the cohort (n=1753).

(Remarks on code availability)
The GitHub page was briefly reviewed, but we did not attempt to run the code yet.

Version 2:

Reviewer comments:

Reviewer #6

(Remarks to the Author)
We thank the authors for largely addressing our previous concerns. Nevertheless, details on the specific weights for

annotators, as well as the final hyperparameters for the various models, would be appreciated (and might be included in supplementary material).

(Remarks on code availability)
The Python code was hosted at https://github.com/su-boussard-lab/GAT-CN. The notebooks were browsed but not executed, and appear generally well-documented.

Open Access This Peer Review File is licensed under a Creative Commons Attribution 4.0 International License, which permits use, sharing, adaptation, distribution and reproduction in any medium or format, as long as you give appropriate credit to the original author(s) and the source, provide a link to the Creative Commons license, and indicate if changes were made.
In cases where reviewers are anonymous, credit should be given to 'Anonymous Referee' and the source.
The images or other third party material in this Peer Review File are included in the article's Creative Commons license, unless indicated otherwise in a credit line to the material. If material is not included in the article's Creative Commons license and your intended use is not permitted by statutory regulation or exceeds the permitted use, you will need to obtain permission directly from the copyright holder.
To view a copy of this license, visit https://creativecommons.org/licenses/by/4.0/

RESPONSE TO REVIEWERS' COMMENTS

Reviewer #2 (Remarks to the Author):

Dr. Saquand and colleagues demonstrate an innovative approach to the use of NLP for extracting symptoms and adverse event monitoring in patients with cancer. Overall, this is an excellent, timely and novel article, and I appreciate the opportunity to review this important work. My comments are included below organized by section.

Introduction:
-The introduction is well written. It might be helpful to the reader to include rates of ACUs with cancer treatment and/or note the percentage of cancer spending on ACU to highlight the scope of the problem.

Response: We thank the reviewer for this suggestion. We have added specific data to illustrate the frequency and impact of ACU during chemotherapy as follows:

"Reported ACU rates vary across cancers and settings: for example, 47% of breast cancer patients experience an ED visit or hospitalization within 180 days of adjuvant chemotherapy [7], while another study found 11.9% hospitalized and 17.1% visiting the ED [8]. Moreover, more than one-third of primary care visits during chemotherapy are for treatment-related side effects, which nearly double the risk of subsequent ED visits or hospitalizations [9]. Together, these examples underscore the substantial burden of chemotherapy-related adverse events on patients and healthcare systems."

[7] Wu, C. H., Quan, M. L., Kong, S., Xu, Y., Cao, J. Q. et al. Acute care use by breast cancer patients on adjuvant chemotherapy in Alberta: demonstrating the importance of measurement to improving quality. Curr. Oncol. 28, 4420–4431 (2021).
[8] Bastedo, S. J., Krzyzanowska, M. K., Moineddin, R., Yun, L., Enright, K. A. et al. A population-based assessment of primary care visits during adjuvant chemotherapy for breast cancer. Curr. Oncol. 24, 90 (2017).
[9] Ruddy, K. J., Van Houten, H. K., Sangaralingham, L. R., Freedman, R. A., Thompson, C. A. et al. Impact of treatment regimen on acute care use during and after adjuvant chemotherapy for early-stage breast cancer. Breast Cancer Res. Treat. 164, 515–525 (2017).

-In the introduction I think it would be helpful to explain a little more deeply what transformer-based architectures and graph neural networks are so that the reader better understands the connection of why the model is novel with the ability to uncover undocumented diagnoses.

Response: We thank the reviewer for this suggestion. In the revised introduction, we now briefly define both transformer-based architectures and graph neural networks and explain why their combination is novel in our context. Specifically, we describe how transformers, such as BERT, capture the contextual meaning of words in clinical text and how GNNs represent information as nodes and edges and aggregate information from neighboring nodes to model relationships between medical concepts. We then link this synergy to the ability of our model to uncover undocumented diagnoses from clinical notes.
Please see the revised part of the introduction below:

“To address these limitations and more accurately capture post-chemotherapy symptoms, we introduce Graph-Augmented Transformer for Clinical Notes (GAT-CN)—a framework that integrates ClinicalBERT [18], a transformer encoder pretrained on clinical text, with graph neural networks (GNNs). The transformer encoder captures rich contextual representations of clinical narratives, enabling nuanced understanding of symptom mentions within patient notes. These representations are incorporated into a heterogeneous clinical graph, where nodes represent patient notes and diagnosis symptom terms derived from an external symptom-class vocabulary, and edges encode contextual and symptom-specific relationships. Using the inductive GraphSAGE algorithm [19], GAT-CN learns node embeddings by aggregating information from neighboring nodes, allowing the model to generalize to new patient notes without reconstructing the entire graph. This integration of contextual language understanding with structured symptom knowledge enables GAT-CN to uncover implicitly documented or contextually linked toxicity symptoms, advancing early detection and supporting proactive monitoring and management of chemotherapy-related toxicity symptoms.”

Methods:
-I would not use the term validation when noting consultation with experts was used. I think it would be more appropriate to say refinement, as validation implies statistical testing, unless Kappa statistics or other validation statistics were compared with physician experts.

Response: Thank you for identifying this terminology concern. We have replaced the term *validated* with *refined* to more accurately reflect that the process involved consultation with experts rather than statistical validation using measures such as Kappa statistics. Please see the revised part of the method (NLP Model Development) below:

“The lists were refined by removing terms absent from the training notes or judged irrelevant by clinicians, while ensuring that each term was specific to a single diagnostic class.”

-The methods state that patients with “blood cancers” were excluded, but the results note that hematopoietic tumors were the most prevalent cancer type and discuss lymphoma patients. The authors should clarify the hematologic malignancies permitted in the study.

Response: We thank the reviewer for identifying this inconsistency in our methods. We have removed the statement “blood cancers” from our methods and clarified our text. Patients with a diagnosis of leukemia were excluded from this analysis due to their explicit exclusion from OP-35 due to leukemia’s high toxicity of treatment and recurrence rates, which result in utilization patterns fundamentally different from other cancers.

Please see the revised part of the method below:
“Following the criteria outlined by the CMS 2023 Measure Updates and Specifications Report Hospital Outpatient Quality Reporting Program [17], we excluded patients with unknown cancer types, patients with leukemia, and those under 18 years of age. Other hematologic malignancies, including lymphoma and myeloma, were retained in the cohort.”

Discussion:
-I would also note that there is limited representation in the patient sample from certain patient races, and some cancer types are missing altogether (multiple myeloma, acute leukemia), which also can limit generalizability.

Response: As noted in response to the previous comment, only leukemia patients were excluded based on the explicit exclusion criteria in OP-35. However, we agree that our sample had limited representation for some cancer types (or even missing) and certain patient races, which may affect generalizability. We have added this as a limitation in the discussion shown below and emphasized the need for future validation of GAT-CN across more diverse populations and cancer types.

"While our study demonstrates meaningful advancements in identifying post-chemotherapy symptoms from clinical notes, several limitations should be acknowledged. First, the study was conducted at a single cancer center with a single holdout test set, limiting generalizability. Nonetheless, successful use of Bio+ClinicalBERT [19], pretrained on notes from a Boston-based health system, suggests broader applicability [45]. Future work should validate GAT-CN on external multi-institutions. … Finally, subgroup performance disparities may arise, particularly across ethnic groups, due to underrepresentation in training data or differences in documentation practices. Future work should expand representation in training cohorts, monitor subgroup-level performance, and adopt hybrid approaches that mitigate bias. While these limitations frame important directions for future work, they do not detract from the contributions and potential impact of GAT-CN in enhancing post-chemotherapy symptom monitoring."

-The Discussion notes that this work builds on prior literature, but I think the Discussion would benefit from a little more direct elaboration on prior NLP work in this area and how this moves the field forward.

Response: This is a great point, and we agree with the reviewer about directly situating our work within prior NLP studies on symptom extraction and clearly articulating what our contribution adds. We added the following paragraph to the discussion section to address this issue.

"These improvements are particularly notable given the limitations of existing methods for symptom extraction. Previous approaches for symptom extraction, including rule-based and transformer-based models, often struggle to capture nuanced clinical associations, particularly in settings with limited annotated data [24]. While transformer models rely on sequence-level patterns, they typically require large datasets to effectively learn contextual associations. Graph-based methods such as TextGCN [25] and MedText [26] have demonstrated the value of leveraging graph structures for textual and biomedical data, but these approaches depend on transductive GCNs, which require a fixed static graph and do not generalize well to unseen data. In contrast, GAT-CN integrates domain knowledge into a heterogeneous graph and applies GraphSAGE, an inductive framework that generates embeddings for new/unseen patient notes without reconstructing the entire graph, thereby enabling more robust symptom extraction for the multi-label task of OP-35 post-chemotherapy symptoms, even with relatively small, annotated datasets."

[24] Patel, P.V. et al. Large language models outperform traditional natural language processing methods in extracting patient-reported outcomes in inflammatory bowel disease. Gastro Hep Adv. 4, 100563 (2025).
[25] Yao, L., Mao, C. & Luo, Y. Graph convolutional networks for text classification. Proc. AAAI Conf. Artif. Intell. 33, 7370–7377 (2019).
[26] Lu, Q., Nguyen, T.H. & Dou, D. Predicting patient readmission risk from medical text via knowledge graph enhanced multiview graph convolution. Proc. 44th Int. ACM SIGIR Conf. Res. Dev. Inf. Retr. 1990–1994 (2021).

Tables and Figures:
-For Supplementary Table 1, I would suggest including a significantly larger number of example terms. This Table is very helpful for giving the reader of sense of what vocabulary is getting flagged.

Response: We appreciate the opportunity to provide readers with a clearer sense of the vocabulary being flagged. We have included a substantially larger set of example terms to illustrate this. See Supplementary Table 1 for the modifications.

-For Supplementary Table 2, the IAA values are fairly modest except for NPFS. Do the authors have a comment or explanation for the more modest IAA for pain and especially for DNVD and anemia?

Response: We appreciate the opportunity to clarify the observed variability in inter-annotator agreement (IAA) across diagnostic labels. The differences in Krippendorff's alpha values reflect inherent challenges in clinical documentation and annotation of these specific conditions:
For Pain, DNVD, and Anemia, the modest agreement is likely from subjective documentation in clinical notes (e.g., "discomfort" vs. "pain") or incomplete information in the notes and variability in documenting severity. In contrast, Neutrophilia, Pneumonia, Fever, Sepsis (NPFS) showed perfect agreement because they rely on objective, consistently documented clinical signs (e.g., "Fever temperature > 38°C"). These symptoms have objective diagnostic criteria, clear laboratory thresholds (e.g., neutrophil count >7.5×10⁹/L), and standardized clinical definitions (e.g., Sepsis-3 criteria). Therefore, documentation is typically explicit and unambiguous in acute care settings. To clarify this, we added the following clarification to the discussion, considering our word limit.

"IAA varied markedly across symptom classes, reflecting the inherent challenges in identifying symptoms from clinical notes. Agreement was highest for symptoms anchored by objective cues (e.g., fever, lab abnormalities) and lower for subjectively described or implicitly documented symptoms (e.g., pain, anemia) [27,28]."

I apologize if I missed it but did not see the code in the files.

Response: We have added the link to the project GitHub in the manuscript and in the submission system.

Please see the added section below:

"Code Availability
The source code and curated dictionary developed for this study are publicly available at https://github.com/su-boussard-lab/GAT-CN, enabling evaluation and use across diverse clinical datasets."

Reviewer #3 (Remarks to the Author):

The manuscript introduces a graph-based method, i.e., GAT-CN, designed to extract chemotherapy-related symptoms from clinical notes. Evaluation demonstrates the method outperformed transformer-based models in multi-symptom labelling. However, there are some issues with the current manuscript.

Major concerns:

1. Graph-based text classification has been studies for years (e.g., the basic idea is to convert or create a graph for a given text, and then encode them with GNNs), yet the paper does not mention any of them as related work. Even for medical texts, there are many existing studies that adopt a similar idea. For example, https://dl.acm.org/doi/10.1145/3404835.3463062.

Response: Thank you for the opportunity to strengthen our work. We have expanded our references to include key prior studies on graph-based text classification. Specifically, we now cite general approaches such as TextGCN [Yao et al., 2019] and biomedical applications such as MedText [Lu et al., 2021], which applied GCNs with UMLS-based multiview graphs to clinical discharge notes. While these studies demonstrate the utility of graph structures for modeling text, they rely on transductive GCNs, which require the entire graph to be fixed at training time. By contrast, our work employs GraphSAGE, an inductive framework that generalizes to unseen patients and notes without reconstructing the graph. Furthermore, our application differs in targeting a novel multi-label classification task, OP-35 post-chemotherapy symptoms, an area not previously addressed in GNN-based clinical NLP. These clarifications have been added to the Discussion to situate our study within the broader literature.
Please see the added section appended to the discussion below:

"These improvements are particularly notable given the limitations of existing methods for symptom extraction. Previous approaches for symptom extraction, including rule-based and transformer-based models, often struggle to capture nuanced clinical associations, particularly in settings with limited annotated data [24]. While transformer models rely on sequence-level patterns, they typically require large datasets to effectively learn contextual associations. Graph-based methods such as TextGCN [25] and MedText [26] have demonstrated the value of leveraging graph structures for textual and biomedical data, but these approaches depend on transductive GCNs, which require a fixed static graph and do not generalize well to unseen data. In contrast, GAT-CN integrates domain knowledge into a heterogeneous graph and applies GraphSAGE, an inductive framework that generates embeddings for new/unseen patient notes without reconstructing the entire graph, thereby enabling more robust symptom extraction for the multi-label task of OP-35 post-chemotherapy symptoms, even with relatively small, annotated datasets."

[24] Patel, P.V. et al. Large language models outperform traditional natural language processing methods in extracting patient-reported outcomes in inflammatory bowel disease. Gastro Hep Adv. 4, 100563 (2025).
[25] Yao, L., Mao, C. & Luo, Y. Graph convolutional networks for text classification. Proc. AAAI Conf. Artif. Intell. 33, 7370–7377 (2019).
[26] Lu, Q., Nguyen, T.H. & Dou, D. Predicting patient readmission risk from medical text via knowledge graph enhanced multiview graph convolution. Proc. 44th Int. ACM SIGIR Conf. Res. Dev. Inf. Retr. 1990–1994 (2021).

2. The method itself is unclear with too many steps. It is very hard to follow. Figures illustrating the logistic connection between various steps could help to understand. For example, it is unclear how the "term nodes" are generated. The architecture of GAT-CN is also unclear, e.g., according to line 177 the BioClinicalBERT embeddings of text is used as node features, however in line 191 there

seems to be a residual connection. Line 132-194 are confusing and it would be better to provide an overview figure of the architecture.

Response: Thank you for this helpful feedback. We agree that our original description of the method could be clearer. In the revised manuscript, we have (i) added a new figure (Figure 3) providing an overview of the GAT-CN architecture and the logical flow of its components, (ii) clarified in the text how the diagnosis term nodes are generated from the OP-35 Diagnosis Vocabulary, and (iii) revised lines 132–194 to improve readability and remove ambiguity regarding the role of Bio+ClinicalBERT embeddings and their integration into GraphSAGE. Together, these updates make the methodological pipeline more transparent and easier to follow.

This is the updated paragraph on the model in the Method section:

“We introduce a symptom diagnostic model, Graph-Augmented Transformer for Clinical Notes (GAT-CN), which integrates Bio+ClinicalBERT [18], with GraphSAGE [19], an inductive graph neural network (GNN), to detect post-chemotherapy symptoms from clinical notes (Figure 3). In this architecture, Bio+ClinicalBERT provides the initial embeddings for patient-note nodes, while GraphSAGE refines these embeddings within a heterogeneous graph that encodes relationships between notes and diagnosis terms. This design allows GAT-CN to capture both local language context and higher-order associations between clinical symptoms. The heterogeneous graph contained two node types: patient clinical note nodes and diagnosis term nodes (generated from the OP-35 Diagnosis Vocabulary, ODV), and two edge types: unweighted edges connecting notes to their associated diagnosis terms, and weighted edges connecting terms that co-occurred in a note, where edge weights reflected positive pointwise mutual information (PPMI).

Patient notes are encoded with Bio+ClinicalBERT [18]; for long notes, the text is chunked and mean-pooled to yield a single vector x_v, which serves as the initial embedding for the corresponding patient-node node ($h_v^0= x_v$). In the GraphSAGE [19] framework, node representations are updated through message passing, where each node aggregates information from its neighbors across depths k. The first layer (k=1) updates the intitialinitial patient-node embedding by integrating the aggregated representations of its immediate neighbor diagnosis-term nodes. Each diagnosis terms nodes are represented by BioWordVec [53] embeddings of dimension d, concatenated with a single scalar encoding the diagnostic class label (an integer between 0 and 3), yielding a (d+1)-dimensional vector for each term. Neighbor embeddings are processed through a convolutional layer, followed by 1D max-pooling and a fully connected (FC) layer to obtain the aggregated neighbor representation $h_{N(v)}^{(1)}$. The updated patient-note embedding is computed via a ReLU-activated FC layer (see Figure 3 for an overview of the GAT-CN architecture at depth k=1). For higher depths (k > 1), the same process is recursively applied as:

$$h_v^k=\mathrm{ReLU}(\mathrm{concat}(h_v^{k-1},h_{N(v)}^k)(W^k)^T+b^k)$$

where $W^{(k)}$ and $b^{(k)}$ denote the learnable weight matrix and bias vector. After k hops, the GraphSAGE-updated patient-note embedding h_v^k—initialized from Bio+ClinicalBERT but not concatenated or residually combined with it—is passed to a linear multi-label head with four sigmoid outputs, yielding the probabilities for each diagnostic class. Only clinical note nodes are used for prediction.”

See Figure 3 for an overview of the model architecture with details in its caption.

3. The experiments are insufficient and some details are missing. For example, it is unknown if GAT-CN has an advantage over other graph-based methods or even rule-based approaches. It would be interesting to see the results with the “term nodes” concatenated as a sequence rather than form a graph as the input. Some details are missing, including the number of nodes/edges for each input.

Response: We thank the reviewer for these valuable comments. Below, we provide our responses to the three points raised:

1. Comparison with other graph-based methods: Our proposed method, GAT-CN, is built on an inductive graph representation learning framework (GraphSAGE), which allows the model to generalize to unseen nodes or entirely new graphs. Many commonly used GNNs operate in a transductive setting, where embeddings are learned for a fixed set of nodes within a static graph, limiting their applicability in our clinical notes setting, where new patients and symptoms frequently appear. Therefore, we believe that a direct comparison to these transductive GNNs would not be appropriate. We have clarified this distinction in the revised Discussion section, in response to Comment #1, to make our design choices more transparent.

2. Comparison with rule-based methods: We have included Random Forest as a baseline, which is an ensemble of decision trees and thus can be considered a representative rule-based method. Each tree applies a series of if–then rules, and the ensemble combines these rules to enhance robustness. We have expanded the description of this baseline in the Methods section to make this connection clearer to the reader. Additionally, we included a simple string matching method using our OP-35 Diagnosis Vocabulary to show the limitations of the simple rule-based methods in symptom extraction.

3. Using “term nodes” as a sequence rather than a graph: We agree this is an important consideration. The “term nodes” in our graph are essentially word embeddings, and concatenating them into sequences corresponds to traditional document representation approaches such as Doc2Vec or sequence-based models. Given the strong adoption of transformer-based models in clinical NLP, we have already included BioClinicalBERT as a representative of this approach, which provides a fair state-of-the-art sequence-based comparison. We have made this connection explicit in the manuscript to clarify that this baseline addresses the reviewer’s point.

4. Number of nodes/edges for each input: We have determined the number of nodes and edges in each depth of graph for GraphSAGE when learning embeddings from neighbors, as follows: “For GraphSAGE, we used a maximal search depth of 2, sampling 150 term-node neighbors and 50 note-node neighbors per layer.”

4. The study design was contradictory, they first claimed that “we excluded patients with unknown cancer types, blood cancers, and those under 18 years of age.” In the result, they reported “Lymphoma tumor patients were more likely to be diagnosed with anemia 267 (n = 166, 28.9%)”. As lymphoma is a type of blood cancer, the method is not convincing and rigorous.

Response: We thank the reviewer for pointing out this inconsistency. We apologize for the unclear wording in the methods. In alignment with OP-35 specifications, we excluded only patients with

leukemia, while other hematologic malignancies (e.g., lymphoma, multiple myeloma) were included in the study cohort. We have revised the methods section to state this explicitly and to ensure consistency with the results. Please see the revised part of the method below:

"Following the criteria outlined by the CMS 2023 Measure Updates and Specifications Report Hospital Outpatient Quality Reporting Program [17], we excluded patients with unknown cancer types, patients with leukemia, and those under 18 years of age.

5. The manuscript also claimed this is a "retrospective cohort study", which is a specific study method in traditional epidemiology. This is not accurate, the study is more like methodology development.

Response: We thank the reviewer for this clarification. We agree that our study is best described as methodology development using retrospective EHR data rather than a traditional retrospective cohort study. We have revised the terminology throughout the manuscript to reflect this distinction and avoid confusion. For example, we revised the beginning of the Methods section, as follows:

"We conducted a methodology development and a validation study using retrospective EHR data."

6. The method focused on detecting cancer therapy-related toxicities, however, a large amount of pre-processing was done to select notes for study. The so-called novel method extracted symptoms only. The relation between cancer therapy and toxicities were established through the logic of "following chemotherapy initiation that are linked to ACU events within 30 days." In this sense, it's not accurate to say the contribution of the method is for detecting cancer therapy-related toxicities. It is only symptom detection, which is similar to NER task in NLP.

Response: We thank the reviewer for this comment. We agree that the model's direct output is detection of symptoms from unstructured clinical notes. However, the novelty of GAT-CN lies not in simple symptom extraction but in its specific design to capture symptoms temporally associated with chemotherapy-induced toxicities. This is achieved by:

Contextual Framework: The analysis is intentionally confined to notes documented within a 30-day window following chemotherapy initiation and preceding an acute care unit (ACU) visit.

Advanced Modeling: Unlike standard NER methods, GAT-CN uses a graph-augmented transformer to model relationships between symptoms, capturing clinically meaningful patterns (e.g., "fever" + "neutropenia") that indicate specific toxicities, which is evidenced by its performance gains over BioclinicalBERT.

We revised the manuscript to clarify this point, making it explicit that the model identifies symptoms temporally *associated* with chemotherapy-induced toxicities, rather than inferring causality. Additionally, we clarified that the novel aspect of our work lies in developing a graph-augmented NLP approach using GraphSAGE, an inductive GNN approach, for symptom extraction, which is a prerequisite for accurately characterizing chemotherapy-related toxicities. Accordingly, we revised the following sections:

Updated sentence in abstract:
"To address this gap, we developed a natural language processing (NLP) model, Graph-Augmented Transformer for Clinical Notes (GAT-CN), designed to extract symptoms temporally

associated with chemotherapy-induced toxicities from clinical notes—including pain, nausea, vomiting, dehydration, diarrhea, anemia, neutropenia, fever, sepsis, and pneumonia."

Updated sentences in discussion:
"In this study, we introduce a novel approach for extracting post-chemotherapy symptoms that are temporally associated with cancer therapy-related toxicities, emphasizing the importance of patient-centered monitoring and management."

Expanded limitations in discussion:
"We focus exclusively on symptom extraction from post-chemotherapy notes. While the model was trained on clinical notes temporally associated with the 30-day ACU period, when treatment toxicities often emerge, GAT-CN detects/classifies symptoms but does not quantify therapy–toxicity associations, which may be implicit in text."

7. The study collected a list of toxicity terms, however, the comparison to a rule-based method was not conducted. The study claimed that they "introduce a novel approach for detecting cancer therapy-related toxicities". But only 4 classes of toxicities were focused on, the mention of toxicities was an exaggeration. What are the numbers of labels for each class?

Response: We thank the reviewer for this comment. We agree that stating our model performs "toxicity detection" was an overstatement. We have revised the manuscript throughout to clarify that the task is symptom extraction for toxicity monitoring. The related changes were determined in our response to Comment #6.

The model was trained on symptoms related to ten OP-35 diagnoses, determined by ICD codes. These were grouped into four overarching classes based on clinical actionability to simplify the clinical workflow. All models were evaluated on these four classes to ensure a consistent comparison. This rationale has been added to the Methods section, as follows:

"The OP-35 measure defines ten post-chemotherapy diagnoses: pain, nausea, vomiting, dehydration, diarrhea, anemia, neutropenia, fever, sepsis, and pneumonia. For the purposes of this study, these ten diagnoses were grouped into four clinically actionable categories to simplify model training and evaluation: (Class 1) Pain; (Class 2) Diarrhea, Vomiting, Nausea, Dehydration (DVND); (Class 3) Anemia; and (Class 4) Neutrophilia, Pneumonia, Fever, Sepsis (NPFS). The diagnoses within each class share overlapping symptom presentations, clinical management strategies, and related comorbidities, providing the clinical rationale for their grouping in this study."

A comparison against a simple rule-based dictionary-matching baseline (flexible matching) was indeed conducted. As expected, the rule-based method achieved a high weighted average recall of 0.99 since notes were pre-selected by symptom keywords but a significantly lower weighted average precision of 0.505 than our model's, 0.780 (p<.001). This highlights the impact of the contextual linguistic features (e.g., negation, historical mentions), which cannot be assessed by dictionary-based matching methods, and demonstrates the value of our contextual NLP approach beyond simple term matching. These results are presented in supplementary materials and reported in the Results section. The following paragraph was also added to the discussion:

"IAA varied markedly across symptom classes, reflecting the inherent challenges in identifying symptoms from clinical notes. Agreement was highest for symptoms anchored by objective cues

(e.g., fever, lab abnormalities) and lower for subjectively described or implicitly documented symptoms (e.g., pain, anemia).[27, 28] This variability also illustrates why simple rule-based methods struggle, as they tend to capture many mentions but conflate nuance, yielding high sensitivity with poor precision.[24,29] In contrast, GAT-CN leverages narrative context and relationships among clinical symptoms, enabling more precise and stable extractions and better handling of co-occurring symptoms within the same note. Taken together, these findings emphasize both the complexity of symptom representation in real-world documentation and the value of graph-augmented, context-aware approaches over rule-based methods."

The number of labels for each class has been determined in Figure 4 for our test set. For example, the test set with EHR labels includes 68 notes with Pain, 47 with DVND, 35 with Anemia, and 53 with NPFS. The whole dataset labels are presented in Table 1.

[24] Patel, P.V. et al. Large language models outperform traditional natural language processing methods in extracting patient-reported outcomes in inflammatory bowel disease. Gastro Hep Adv. 4, 100563 (2025).
[27] Du, N., Chen, K., Kannan, A., Tran, L., Chen, Y., and Shafran, I. Extracting Symptoms and their Status from Clinical Conversations. In Proceedings of the 57th Annual Meeting of the Association for Computational Linguistics (pp. 915–925). Association for Computational Linguistics. (2019)
[28] Oommen C., Howlett-Prieto Q., Carrithers MD., Hier DB. Inter-rater agreement for the annotation of neurologic signs and symptoms in electronic health records. Frontiers in Digital Health. 5:1075771 (2023).
[29] Hernandez-Boussard T., Tamang S., Blayney D., Brooks J, Shah N. New paradigms for patient-centered outcomes research in electronic medical records: an example of detecting urinary incontinence following prostatectomy. eGEMs. 4(3):1231 (2016).

8. It's not clear how the evaluation was done. Is it evaluated based on span match, exact match or flexible match? There is an inconsistency in evaluation metrics AUROC and F1 were used in various instances, why?

Response: We thank the reviewer for this comment and apologize for the lack of clarity. We clarified the evaluation methodology and metric definitions in the Methods section under "Model Evaluation" as follows:

"Diagnosis symptom extraction was evaluated at the patient-note level. A symptom was considered correctly identified if the model detected at least one mention corresponding to the annotated symptom (flexible match), rather than requiring exact span matching, to account for variability in clinical documentation. Model performance was assessed per symptom class using Receiver Operating Characteristic (ROC) and Precision-Recall (PR) curves. We reported weighted averages of the Area Under ROC (AUROC) curve and Area Under PR Curve (AUPRC) to summarize performance across all prediction thresholds. In addition, we reported weighted averages of precision, recall, and F1-score at a decision threshold of 0.5. The F1-score, the harmonic mean of precision and recall, is particularly informative in clinical settings as it balances sensitivity to true positives with minimization of false alarms.

To evaluate performance in the multi-label setting, where a single patient's notes can contain multiple correct labels, we used three complementary metrics: (1) Hamming loss (HL): the fraction of incorrectly predicted labels out of the total number of labels across all samples; (2) Subset

accuracy (SA): the strictest measure, reflecting the percentage of notes for which the entire predicted label set exactly matches the true set; and (3) Sample-averaged F1-score: the F1-score computed for each note and then averaged across all notes."

"We used the sample-averaged F1-score for subgroup analysis to assess performance variations across patient characteristics and to capture the model's ability to identify all relevant symptom classes per note, reflecting joint multi-label performance rather than independent class-based metrics like AUROC. This metric provides a balanced summary of precision and recall, offering more interpretable results than stricter measures such as HL or SA."

Error analysis is desired. For example:
what caused error in the results of IAA: "The overall IAA across the test set varied by class, with perfect agreement for NPFS (IAA = 1.0), fair agreement for Pain (0.59) and DNVD (0.49), and lower agreement for Anemia (0.26)." what caused errors in evaluation: "GAT-CN had a HL of 0.236 298 (95% CI 0.218–0.253), indicating that on average, approximately 23.6% of all labels were predicted incorrectly." Reporting of results need more details, for example, what's the percentage----- "Specifically, they detected 7 patients with Pain, 1 with Anemia, 4 with symptoms related to DVND, and 4 with diagnoses linked to NPFS." What's the impact of the percentage?

Response: We thank the reviewer for this insightful suggestion. We extended our Discussion section by adding the following error analyses:

Error analysis for IAA and multi-label performance:
"IAA varied markedly across symptom classes, reflecting the inherent challenges in identifying symptoms from clinical notes. Agreement was highest for symptoms anchored by objective cues (e.g., fever, lab abnormalities) and lower for subjectively described or implicitly documented symptoms (e.g., pain, anemia).[50,51] This variability also illustrates why simple rule-based methods struggle, as they tend to capture many mentions but conflate nuance, yielding high sensitivity with poor precision.[15,52] In contrast, GAT-CN leverages narrative context and relationships among clinical symptoms, enabling more precise and stable extractions and better handling of co-occurring symptoms within the same note. Taken together, these findings emphasize both the complexity of symptom representation in real-world documentation and the value of graph-augmented, context-aware approaches over rule-based methods."

Error analysis for multi-label setting:
"In the multi-label setting, the model achieved a HL of 0.236 and SA of 0.311, indicating that despite some label errors, GAT-CN effectively captured multiple co-occurring symptoms jointly—a task inherently more difficult than single-label prediction. Error analysis revealed that misclassifications primarily stemmed from ambiguous or implicit symptom mentions, overlapping symptom categories, and sparse documentation of certain symptoms such as anemia. In the graph component, highly connected or sparsely linked nodes occasionally propagated irrelevant information, leading to confusion across co-occurring symptom classes. These factors highlight the inherent complexity of multi-label symptom extraction from clinical text and point to areas for refining contextual and graph representations using a graph-attention mechanism in future work."

Regarding reported results with percentages, we agree that reporting only absolute counts without context can be misleading. Our intention was to demonstrate that our model identified clinically

relevant symptoms that were missed by structured EHR codes. We have revised the results by adding their percentages relative to our test set size to emphasize that these numbers may represent a small but critical subset of patients where our NLP method provided additional clinical insight.

“Additionally, both the annotators and the model identified diagnoses for patients that were absent from the structured EHR. The annotators revealed 11 additional patients for Pain (10.4%), of which GAT-CN correctly identified 7 (63.6%); 10 with DVND (9.4%), of which 4 (40%) were identified; 10 with Anemia (9.4%), of which 1 (10%) was identified; and 7 with NPFS (6.6%), of which 4 (57.1%) were identified.”

Reviewer #4 (Remarks to the Author):

Summary
This paper proposed a natural language processing model to extract chemotherapy-related symptoms from clinical notes to integrate EHR information with unstructured clinical notes. This model demonstrates better performance in multi-symptom labelling compared to transformer models.
Comments:

1. The current evaluation relies primarily on a single holdout test dataset. The absence of additional external or diverse datasets might limit insights into the generalizability of the GAT-CN model across various clinical settings or institutions.

Response: Thank you for this thoughtful comment. We agree that the reliance on a single-center cohort and a single holdout test set may limit generalizability and robustness. Our goal in this study was primarily methodological development and proof-of-concept evaluation of GAT-CN. To mitigate overfitting risks, we used a holdout design with strict patient-level separation between training, validation, and test cohorts. In the revised manuscript, we have updated the Discussion section to explicitly acknowledge this as one combined point and to emphasize the need for future external validation on independent datasets from multiple institutions to confirm robustness and transportability of GAT-CN.

Updated paragraph in the Discussion:
“While our study demonstrates meaningful advancements in identifying post-chemotherapy symptoms from clinical notes, several limitations should be acknowledged. First, the study was conducted at a single cancer center with a single holdout test set, limiting generalizability. Nonetheless, successful use of Bio+ClinicalBERT, pretrained on notes from a Boston-based health system, suggests broader applicability. Future work should validate GAT-CN on external multi-institutions. Second, variability in clinical language may result in inconsistencies in symptom documentation, potentially leading to misinterpretations of patient outcomes [46] [47]. However, our graph-based framework mitigates this by linking related symptoms to validated diagnosis classes using embedding vectors, which reduces the impact of lexical variation. Moreover, because GAT-CN employs GraphSAGE, it enables inductive embeddings for unseen notes, potentially enhancing generalizability across institutions.”

2. Currently, the model's performance analysis is primarily reported using the F1 metric. Including additional sensitivity analyses, such as precision-recall curves, ROC curves, or decision threshold

analyses, would offer deeper insights and further contextualize the model's performance, particularly regarding its sensitivity and specificity trade-offs.

Response: Thank you for this suggestion. We would like to note that ROC and precision–recall curves are already included in the Supplementary Materials (Supplementary Figure 1), where they illustrate model performance across the four post-chemotherapy diagnostic classes and provide additional context on sensitivity–specificity and precision–recall trade-offs.

3. While the paper benchmarks the GAT-CN model against established transformer models, it should also include comparisons against newer state-of-the-art transformer models designed specifically for clinical text, such as Clinical-Longformer/BigBird and BioClinical ModernBERT.

Response: We thank the reviewer for this suggestion. In this study we compared GAT-CN against strong clinical/biomedical transformer baselines (Bio+ClinicalBERT and BioMed RoBERTa) that are widely used in clinical NLP. To ensure consistent comparisons, our preprocessing pipeline segmented notes into ≤512-token chunks, each processed individually before aggregating embeddings. This design aligned with the input limitations of the BERT-based baselines, but it also meant that long-context models such as Clinical-Longformer and BigBird could not fully exploit their extended sequence capacity.

We did experiment with the Longformer model under this segmentation framework, but because it was forced into the same chunked setup as BERT-style models, its performance was modest: AUROC of 773 (0.751 - 0.797), AUPRC of 0.745 (0.722-0.772), HL of 0.278 (0.256 - 0.300), and SA of 0.273 (0.235 - 0.318).

We also emphasize that GAT-CN's graph augmentation is model-agnostic and could be combined with long-context transformers in subsequent experiments for lengthy notes to further improve performance. We have added a statement to the discussion acknowledging this omission and plan to evaluate these models in future work.

We added the following paragraph to the discussion:

"We segmented notes into ≤512-token chunks to align with standard transformer baselines (e.g., Bio+ClinicalBERT, BioMed RoBERTa), ensuring fair comparison but limiting potential benefits from long-context models such as Clinical-Longformer or BigBird. As a model-agnostic framework, GAT-CN can readily incorporate these architectures in future work to capture longer contextual dependencies in clinical text."

4. The supplementary materials lack detailed information regarding model training processes, specifically describing hyperparameter tuning procedures, parameter selection justifications, and including comprehensive training and evaluation loss curves.

Response: Thank you for this important comment. Training and validation losses were monitored throughout all experiments to guide hyperparameter tuning and early stopping, and the final

parameters were reported in the manuscript. As convergence behavior was stable across runs and final models were selected at the point of lowest validation loss, we have summarized this process in the Supplementary Materials by avoiding reproducing multiple similar curves.

Paragraph added in the Supplementary Materials:
“Hyperparameter Selection and Training Dynamics
We tuned hyperparameters empirically through a limited search on the validation set. Because GAT-CN is computationally intensive to train, performing k-fold cross-validation or exhaustive grid search was not feasible. Instead, we evaluated a small set of configurations for learning rate, dropout, batch size, and GraphSAGE neighbor sampling, selecting the final settings that consistently minimized validation loss while maintaining stable training behavior. Training and validation losses were monitored during all experiments, and convergence was stable across runs. For GraphSAGE, we tested different neighbor sampling ranges and kernel widths, choosing the configuration that balanced predictive performance with computational efficiency.”

5. In the methods section, the manuscript does not describe the computational resources of deploying this model, including detailed metrics about training time, hardware requirements, resource utilization, and scalability.

Response: We thank the reviewer for this valuable suggestion. We agree that reporting computational resources is important for transparency and reproducibility. While we did not systematically benchmark training efficiency or scalability, we have added a description of the computing environment and approximate training times in the revised Methods section for GAT-CN.

Added paragraph in Methods:
“Computational Resources
Model training and evaluation were performed primarily on a machine with a 10-core CPU (2.4 GHz) and 32 GB of RAM, where the Bio+ClinicalBERT and GraphSAGE components were trained jointly. Depending on hyperparameter settings, average run times per epoch ranged between 1 and 2 hours, with early stopping typically reached after 6–8 epochs. As a proof-of-concept, we also tested training on an Apple M1 GPU, where per-epoch runtime was substantially reduced (~9 minutes per epoch), illustrating that modern GPUs can dramatically accelerate training. These observations indicate that GAT-CN can be trained efficiently on standard CPU hardware and that GPU acceleration can facilitate larger-scale validation across datasets.”

6. While the manuscript adequately addresses lower inter-annotator agreement scores for the Anemia classification, it lacks suggested strategies to enhance annotation reliability.

Response: Thank you for this valuable comment. We agree that our original discussion did not sufficiently address potential strategies to enhance annotation reliability. In the revised manuscript, we discussed the reasons for the variability of IAA and added a concise sentence outlining three possible approaches for future work: clearer annotation guidelines, calibration sessions among annotators, and consensus adjudication of disagreements.

Added in the Discussion:
“To improve annotation reliability, future work should include clearer annotation guidelines, calibration among annotators, and consensus adjudication of disagreements [28].”

7. The discussion did not address subgroup performance disparities, particularly regarding ethnicity, offering detailed hypotheses for observed disparities and proposing concrete, actionable strategies to improve model fairness, such as diversifying training data, targeted fine-tuning, or fairness-aware algorithmic adjustments.

Response: This is an important observation. We agree that subgroup performance disparities, particularly across ethnicity, warrant explicit consideration. In the revised manuscript, we have added a sentence in the Discussion acknowledging potential sources of such disparities (e.g., underrepresentation in training data or systematic differences in documentation) and outlining concrete strategies for improvement. Specifically, we note the importance of expanding representation in training cohorts, routinely evaluating model performance across patient subgroups, and leveraging hybrid approaches that integrate structured and unstructured data to mitigate documentation-related biases.

Added text in Discussion:
“Finally, subgroup performance disparities may arise, particularly across ethnic groups, due to underrepresentation in training data or differences in documentation practices. Future work should broaden cohort diversity, monitor subgroup-level performance, and adopt fairness-aware or hybrid approaches integrating structured and unstructured data to mitigate documentation- and data-related biases.”

8. Code Availability is not provided or mentioned.

We thank the reviewer for pointing this out. We have added a Code Availability section in the revised manuscript:

“Code availability
The source code and curated dictionary developed for this study are publicly available at https://github.com/su-boussard-lab/GAT-CN, enabling evaluation and use across diverse clinical datasets.”

Minor comments:
Line 227-229, it is ambiguous to state that ‘we benchmarked it against four transformer models’, as only three transformer models were listed and the traditional Radom Forest classifier is not transformer model.

Response: We have revised the text to read *“three”* instead of *“four.”*

Reviewer #4 (Remarks on code availability):
Code availability is not mentioned/provided in the manuscript.

We addressed this above in response to the previous comment (#8)

Reviewer #5 (Remarks to the Author):

I co-reviewed this manuscript with one of the reviewers who provided the listed reports. This is part of the Nature Communications initiative to facilitate training in peer review and to provide appropriate recognition for Early Career Researchers who co-review manuscripts.

RESPONSE TO REVIEWERS' COMMENTS

Reviewer #2 (Remarks to the Author):

The authors have sufficiently addressed all of my prior comments. Thank you for the opportunity to review this work.
Response: We thank the reviewer for their time.

Reviewer #4 (Remarks to the Author):

The authors have addressed previous comments. The author added more information about ACU and transformer/GAT-CN frameworks in the introduction section.
The paper now includes the single holdout test evaluation as a limitation in the discussion
The authors explain why they did not select more updated BERTs such as BioClinical ModernBERT for the study.
The authors include the computational information in the method section.
There are no other major or minor comments to this paper.
Response: We thank the reviewer for their time.

Reviewer #5 (Remarks to the Author):

I co-reviewed this manuscript with one of the reviewers who provided the listed reports. This is part of the Nature Communications initiative to facilitate training in peer review and to provide appropriate recognition for Early Career Researchers who co-review manuscripts.
Response: We thank the reviewer for their time.

Reviewer #6 (Remarks to the Author):

This manuscript describes an NLP GAT-CN model that is designed to extract symptoms temporally associated with chemotherapy-induced toxicities from clinical notes, towards predicting acute care utilization (ACU) events. In particular, GAT-CN embeds patient notes with the Bio+ClinicalBERT transformer encoder, which are then linked to diagnosis-related terms in a (pre)trained graph model. This was found to outperform transformer-only approaches.

The early prediction of ACU risk is an important and under-explored challenge. However, there may be a number of issues with the present form of the manuscript, in particular relating to the exact task definition for GAT-CN:

1. In the Results section, it is stated that the target cohort comprised 1753 patients who received acute care within 30 days of initiation of chemotherapy. However, in practical application for the proactive prediction of ACU events (as implied in the Introduction), the 30-day acute care status of patients would be necessarily unknown. If so, the applicability of the cohort to the intended task scenario might thus be further justified.

Response: We thank the reviewer and agree that ACU status would not be known at the time of chemotherapy initiation in a real-world, prospective setting. The goal of this study is not to predict ACU at treatment initiation but rather to extract and characterize chemotherapy-related toxicity symptoms from unstructured clinical notes that occur during routine care and may precede subsequent ACU events. The cohort restriction to patients who experienced ACU within 30 days allows us to rigorously assess whether relevant toxicity symptoms were documented in clinical notes prior to the ACU event and whether such symptoms are missing from structured EHR data. Importantly, all model inputs and analyses are limited to documentation available before the ACU event, consistent with the intended real-world use case of symptom monitoring rather than ACU prediction.

We have revised the last paragraph of the introduction section to clarify this distinction and to explicitly state the proposed model focus is on documents available prior to ACU to identify post-chemotherapy-related symptoms.

“To address these limitations and more accurately capture post-chemotherapy symptoms **documented during routine care**, we introduce Graph-Augmented Transformer for Clinical Notes … The transformer encoder captures rich contextual representations of clinical narratives, enabling nuanced understanding of symptom mentions within patient notes **available prior to downstream acute care events**. … This integration of contextual language understanding with structured symptom knowledge enables GAT-CN to uncover implicitly documented or contextually linked

toxicity symptoms, **which may precede acute care utilization,** advancing early detection and supporting proactive monitoring and management of chemotherapy-related toxicity symptoms **rather than direct prediction of acute care events.**"

2. It is then stated that of the ACU patients (assumed to be the full cohort of 1753 patients in the target cohort), 95 experienced an ACU event during their observation period (defined as t_1 - t_0 in the problem definition in Figure 2, where t_f - t_0 = 30 days). It might be clarified as to whether the remaining (1753 - 95) patients had (one or more) ACU events after t_1 but before t_f (i.e. after the observation period), and whether the GAT-CN model is supposed to predict ACU risk only for this post-observation period (because the required EHR data is collected during the observation period).

Response: We apologize for the confusion raised by this statement: "Of the ACU patients, 401 were seen and discharged from an ED, 1,257 were admitted as inpatients to the hospital, and 95 experienced an ACU event during their observation period." To clarify, all 1,753 patients in the target cohort experienced at least one ACU event, defined as an ED visit or inpatient hospitalization, within 30 days of chemotherapy initiation. The 95 patients experienced both an ED visit and an inpatient hospitalization during the observation period. We have revised the sentence as follows:

"Among the 1,753 patients who experienced at least one ACU event within 30 days of chemotherapy initiation, 401 had an ED visit without subsequent admission, 1,257 were admitted as inpatients, and 95 experienced both ED and inpatient during the observation period."

3. In the Diagnosis Vocabulary and Note Annotation subsection, it is stated that the training and validation sets were labeled using patient OP-35 diagnosis codes in the EHR data, but the test set was manually annotated by four annotators. This difference in annotation between the training/validation and test sets might be explained - was annotation by diagnosis code inapplicable for the test set?

Response: We intentionally used different labeling strategies to balance scalability and evaluation rigor. Diagnosis code–based labels enabled scalable supervision for training and validation, reflecting real-world EHR constraints. Manual annotation was used for the held-out test set to provide a higher-fidelity reference standard for evaluating symptom extraction from free-text notes.

We revised the mentioned subsection as follows:

“Although diagnosis code-based labels were also available for the test set, we intentionally relied on manual annotation by four independent annotators for the held-out test data to establish a higher-fidelity reference standard. This annotation also captures symptoms that may be absent or incompletely represented in the EHR data, enabling more robust evaluation.”

4. For the diagnostic-related terms, it might be clarified as to whether and how (possibly non-standard) abbreviations and misspellings were handled.

Response: Thank you for this question. Diagnostic-related terms were not matched using strict exact string matching alone. Prior to graph construction, clinical notes underwent standard text normalization, including lowercasing, punctuation removal, and tokenization. Diagnostic-related terms were derived from a curated symptom-class vocabulary that included common clinical abbreviations and synonymous variants (e.g., ‘n/v’ and ‘n&v’ for nausea and vomiting, ‘cbc’ for complete blood count).

In addition, because GAT-CN operates on contextualized embeddings generated by Bio+ClinicalBERT rather than surface string matches, semantically similar variants, misspellings, and shorthand expressions are mapped to nearby representations in embedding space and can be associated with diagnostic-related nodes through the learned graph structure. This reduces sensitivity to non-standard spellings and abbreviations commonly observed in clinical notes.

We revised the method section to clarify this issue as follows:
“To address the inherent variability in clinical documentation, we incorporated common abbreviations and synonymous variants into the symptom vocabulary and leveraged contextualized embeddings, rather than strict string matching, to support robust symptom identification.”

5. The specific data used to develop and validate the Bio+ClinicalBERT transformer encoder, as well as the GraphSAGE-trained graph model, might be clarified. The Model Development subsection stated that approximately 6% of the cohort was held out for testing, with 20% of the remaining data used for validation, but it is not fully clear as to whether the individual transformer and graph components of GAT-CN were trained/fine-tuned.

Response: We revised the model development section to clarify how we used data for training and fine-tuning both Bio+ClinicalBERT and GNN, as follows:

“GAT-CN was trained jointly in an end-to-end manner. Bio+ClinicalBERT was initialized with publicly available pretrained weights and fine-tuned during training, while the

GraphSAGE component was randomly initialized and trained simultaneously. Model optimization was performed exclusively on the training split, with validation used for early stopping and hyperparameter tuning, and the test set held out entirely for final evaluation.”

6. In the Discussion section, transductive GCNs were critiqued as requiring a fixed static graph that do not generalize to unseen data. However, the GraphSAGE framework appears to also yield a fixed static graph, for a given set of training data; it might be clarified as to whether this understanding is correct.

Response: Thank you for this helpful clarification. We revised the Discussion section to clarify the distinction between transductive GCN approaches and the inductive nature of GraphSAGE used in GAT-CN.

Revised the paragraph in the Discussion section:

“Although GraphSAGE is trained on a graph constructed from the training data, it learns aggregation functions that generalize to unseen notes based on their features and local graph structure, without requiring retraining or full graph reconstruction.”

7. In the Model Evaluation subsection, it is stated that the ground truth for model performance evaluation was generated by manual annotation on patients' clinical notes (obtained before t_1, from Figure 2). However, if the intended objective is proactive prediction, the relevant ACUs would arise only after the notes were recorded (i.e. after t_1, before t_f). If so, it might be clarified as to whether the annotation using the clinical notes (obtained before t_1) would reflect future patient ACU (after t_1).

Response: Thank you for raising this point. As identified above, we clarified that ACU is not a prediction target in this study. Manual annotation reflects symptom presence in notes documented prior to time t_1 and does not represent future ACU outcomes.

We revised the mentioned sentence as follows:
“To evaluate model performance, clinical notes documented prior to ACU were manually annotated to establish ground truth for symptom presence, rather than to predict subsequent ACU.”

8. In the Model Evaluation subsection, it is stated that weighted voting was used to finalize labels. Details about how the relevant weights were assigned might be stated.

Response: We have revised the sentence to clarify weighted voting, as follows:

“Annotators with formal oncology or advanced clinical training were assigned higher weights than annotators with less clinical experience.”

9. In the Model Evaluation subsection, benchmarking against existing transformer-based models and baseline approaches was described. An ablation experiment with different embeddings as input to the graph model might be appropriate, to support the chosen Bio+ClinicalBERT embedding.

Response: We thank the reviewer for this thoughtful suggestion. Our ablation experiments were designed to isolate and evaluate the contribution of the inductive graph component, rather than to compare or optimize alternative text embedding models. We therefore used Bio+ClinicalBERT as a strong and widely adopted clinical text encoder and focused on benchmarking transformer-only baselines against their graph-augmented counterparts.

As noted in the Limitations section, GAT-CN is model-agnostic with respect to text embeddings, and alternative encoders, including models designed for longer clinical contexts, can be readily substituted within the framework. Exploring different embedding choices would be a valuable extension of this work but was beyond the scope of the current study.

10. For the Random Forest baseline approach, it might be clarified as to whether hyperparameter optimization had been attempted.

Response: Key hyperparameters, including the number of trees and maximum tree depth, were optimized for the Random Forest baseline using the training set, with the validation set used for model selection. The test set was held out and used only for final evaluation. We have clarified this in the Model Evaluation subsection as follows:

“For the random forest baseline, hyperparameters were tuned on the training set using validation-based model selection.”

11. In Figure 3, it is stated that each diagnosis-term node combines a 200-dimensional BioWordVec embedding, with a 1-dimensional diagnosis-symptom class encoding. It might be commented as to whether the 1-dimensional encoding is sufficient, especially given the relatively low dimensionality of the cohort (n=1753).

Response: We thank the reviewer for this insightful question. The 1-dimensional diagnosis-symptom class encoding is intended as a lightweight indicator of class

membership rather than a rich semantic representation. The primary representational capacity of diagnosis-term nodes is provided by the 200-dimensional BioWordVec embeddings, which capture semantic similarity among clinical terms, and by neighborhood aggregation within the graph. The symptom-class indicator anchors diagnosis terms to curated symptom categories while avoiding unnecessary model complexity. Given the relatively modest cohort size, we chose a low-dimensional encoding to avoid overparameterization.

We have clarified the role of this feature in the Figure 3 description as follows:

“The 1-dimensional diagnosis-symptom class encoding serves as a lightweight indicator of curated symptom categories, while semantic information is primarily captured by the BioWordVec embeddings and graph-based aggregation.”

RESOPONSE TO REVIEWERS' COMMENTS

Reviewer #6 (Remarks to the Author):

We thank the authors for largely addressing our previous concerns. Nevertheless, details on the specific weights for annotators, as well as the final hyperparameters for the various models, would be appreciated (and might be included in supplementary material).

Response: We thank the reviewer for this suggestion. We have revised the Methods section to explicitly report the annotator weighting scheme used during the consensus labeling process (0.40 for the oncologist, 0.30 for the medical doctor, and 0.15 for each of the remaining annotators).

Regarding hyperparameters, we included additional architectural details and hyperparameters in the Supplementary Section on Hyperparameter Selection and Training Dynamics, which we refer to in the manuscript.

Added paragraph to supplementary:

Transformer-based models, RoBERTa-base and Longformer-base, utilized pre-trained weights with architecture-specific configurations. Specifically, RoBERTa consisted of 12 transformer layers with a hidden size of 768 and 12 attention heads. The Longformer adopted the same base architecture but employed a sliding-window attention mechanism with a window size of 512.

Reviewer #6 (Remarks on code availability):

The Python code was hosted at https://github.com/su-boussard-lab/GAT-CN. The notebooks were browsed but not executed, and appear generally well-documented.

Response: We thank the reviewer for taking the time to review the code.
